# Identification and characterization of yeast SNF1 kinase homologs in *Leishmania major*

**DOI:** 10.3389/fmolb.2025.1567703

**Published:** 2025-03-24

**Authors:** Gaurav Shoeran, Namrata Anand, Upninder Kaur, Kapil Goyal, Rakesh Sehgal

**Affiliations:** ^1^ Department of Medical Parasitology, Postgraduate Institute of Medical Education and Research, Chandigarh, India; ^2^ Department of Virology, Postgraduate Institute of Medical Education and Research, Chandigarh, India

**Keywords:** *Leishmania major*, SNF1, CRISPR-Cas9, stress adaptation, ribosomes

## Abstract

**Background:**

Sucrose Non Fermenting1 (SNF1) constitutes a family of protein kinases conserved in eukaryotes, plants, and fungi. SNF1 has been known to play a crucial role in stress adaptation and metabolism, enabling organisms to respond to changing environmental conditions. Initially identified in yeast, SNF1 is essential for shifting from the primary carbon source, glucose, to secondary carbon sources like sucrose. Homologs of this protein family were identified in *Leishmania major*, a protozoan parasite and we aimed to determine their role in this parasite.

**Methods:**

In the present study, we identified the putative homologs of SNF1 kinase in *L. major* and knock out strains were prepared using the CRISPR-Cas9 knock-out strategy. The developed strains were evaluated for their growth, characteristics, protein expression and ultra structural changes *in vitro* and virulence in a mouse model.

**Results:**

One of the strain named N2, was found to be completely avirulent and showed limited growth, lack of glycosomes and had a fewer mitochondria with deformed cristae. The N2 strain failed to produce infection in mice when compared to WT mice. Proteome analysis revealed an increase in ribosomal proteins in the N2 strain, highlighting the role of ribosomes in stress adaptation.

**Conclusion:**

The essentiality of this gene for developing infections in mice underscores its potential in the development of future antileishmanial therapies and live attenuated strains.

## 1 Introduction

Living organisms must adapt to ever-changing environmental conditions for survival. Both unicellular and multicellular organisms face diverse types of stresses, making stress adaptation essential for their vitality. This adaptation requires cells to alter their metabolism to better survive in new environments. Under stress, energy homeostasis is adjusted to suit the changing conditions (Guan et al., 2017).

Sucrose Non-Fermenting 1 (SNF1) and AMP-Activated Protein Kinase (AMPK) are a family of protein kinases that maintain energy homeostasis and stress adaptation in both multicellular and unicellular organisms (Hedbacker and Carlson, 2008; Crozet et al., 2014). SNF1 kinase exists as a heterotrimer with a catalytic α subunit and regulatory β and γ subunits (Amodeo et al., 2007). These proteins have been characterized in multicellular mammals, plants, and unicellular fungi. In plants, this family of serine-threonine protein kinases is known as SNF1-related protein kinases 1 (SnRK1) (Crozet et al., 2014). SNF1 kinase was first identified in *Saccharomyces cerevisiae* for its role in releasing glucose repression (Wilson et al., 1996). Catabolite repression is a phenomenon where a preferred carbon/energy source turns off the catabolism machinery of secondary sources, observed in both prokaryotes and eukaryotes (Caligaris et al., 2023). In the absence of a glucose source, yeast mutants lacking SNF1 or having a mutated copy are unable to utilize alternative energy sources such as sucrose, galactose, maltose, melibiose, and ethanol (Jiang and Carlson, 1996). During glucose derepression, many genes are altered in their expression by the upstream SNF1 kinase. Under glucose limitation, yeast changes the expression of numerous genes to feed intermediates to the TCA cycle, glycolysis, and other essential metabolic pathways in the cell (Hedbacker and Carlson, 2008). Besides glucose derepression, SNF1 kinase also plays roles in adapting to various other cellular stresses (Hong and Carlson, 2007).

AMPK is a homolog of SNF1 in mammals, acting as an energy sensor (Hardie et al., 2012). Like in yeast, AMPK forms a trimeric complex with catalytic α, regulatory β, and γ subunits (Xiao et al., 2011). Cells sense the ATP/AMP and AMP/ATP ratios and activate AMPK under low ATP levels. AMPK activation is primarily triggered by declining energy levels induced by exercise (Spaulding and Yan, 2022), ischemia (Cai et al., 2022), heat shock (Wang et al., 2010), and oxidative stress (Wu and Wei, 2012), leading to the activation of catabolic processes and inhibition of anabolic processes to increase ATP levels.

Leishmaniasis is a disease caused by *Leishmania*, a protozoan parasite with a digenetic life cycle infecting mammals and insect vectors (Tom et al., 2024). *Leishmania* parasite exist in three different states, *Leishmania donovani* causes visceral leishmaniasis which affects reticuloendothelial organs like spleen and liver. *Leishmania major* causes infection in skin dermis (cutaneous leishmaniasis) or mucocutaneous membranes (mucocutaneous leishmaniasis) (Remadi et al., 2017). Currently, no vaccine is available, but various treatment approaches are in different phases of study (Kim et al., 2023; Sharma et al., 2023; Kadayat et al., 2024).

Stress adaptation is crucial in *L. major* for the development of metacyclic parasites (Matte et al., 2021). During its life cycle, *L. major* must cycle between different hosts and a wide range of temperature and nutritional conditions, highlighting the importance of metabolic adaptation for its survival. Therefore, in this study, putative homologs of the yeast SNF1 kinase were identified in *L. major*, and their role in stress adaptation was studied by developing and characterizing knock-out (KO) strains and compared with wild type (WT) strains. One of the KO strains developed showed limitations in nutritional requirements and loss of infection in mice and was further characterized in detail. A comparative whole proteome analysis was also performed to identify changes in protein expression to confirm the KO strain characteristics.

## 2 Materials and methods

### 2.1 Bioinformatics

Homologs of the *S. cerevisiae* SNF1 kinase were identified in *L. major* using PBLAST analysis. Proteins with a score of more than 200 and containing the STKc_AMPK_alpha domain were selected. Multiple Sequence Alignment (MSA) was performed to identify homologous regions. A phylogenetic tree was constructed using PhyML based on the protein sequences.

### 2.2 Parasite maintenance

*L. major* strain 5ASHK was kindly provided by Professor Bhaskar Saha from NCCS Pune, India. The strain was cultured in RPMI1640 media (#50-020-PB, Corning) supplemented with 10% heat-inactivated fetal bovine serum (HIFBS) (#RM9951, HIMEDIA) and penicillin and streptomycin (#15140122, Gibco) as antibiotic agents. *Leishmania* promastigote culture was also maintained on 10% rabbit blood agar plates containing RPMI1640 medium, 10% HIFBS, and an additional RPMI1640 as an overlay with antibiotic supplementation. The WT avirulent strain was developed by continuously maintaining the promastigote stage of *L. major* in liquid culture medium for 1 year. In contrast, the virulent strain was maintained by inoculating the parasite into mice footpads, harvesting the amastigotes, and converting them to promastigotes after 2 months. Antibiotics hygromycin (#10687010, Gibco) was used at 50 μg/mL, G-418 (# 10131035, Gibco) at 40 μg/mL, and puromycin (# A1113803, Gibco) at 40 μg/mL.

To maintain the virulence of the promastigotes, freshly differentiated promastigotes from amastigotes are usually collected and maintained so for about 20 passages. Virulence is known to decrease with serial passages over time (Segovia et al., 1992; Moreira et al., 2012). Therefore, in the present study for generating KO’s, virulent passage three (P3) parasites were used to create Cas9-expressing parasites. All KO strains developed were at passage four to five (P4-P5). The WT strain was used as a control in infection experiments, and the attenuated WT avirulent strain was also used to assess loss of virulence (promastigotes passages *in vitro* for more than 1 year). Only KO strains with differential growth requirements were chosen for virulence studies.

### 2.3 Animals

All experiments were conducted in accordance with the Institute Animal Ethics Committee guidelines at PGIMER, Chandigarh, India, under approval notice 100/99/IAEC/690. Inbred BALB/c mice were purchased from IISER Mohali, India, and maintained in a specific pathogen-free Biosafety Level 2 facility at PGIMER, Chandigarh, India. New Zealand white rabbits were provided by the animal house at PGIMER, Chandigarh, India. Rabbit blood was collected through the cardiac puncture, defibrinated, and used immediately for preparing blood agar plates and slants.

### 2.4 Generation of KO strains

KO strains were developed using the CRISPR-Cas9 strategy (Beneke et al., 2017), with some modifications. Briefly, 50 µg of the Cas9-expressing plasmid pT007 (a kind gift from Dr. Eva Gluenz, University of Oxford, UK) was electroporated into *L. major* promastigotes using the Neon electroporation system (# NEON1SK, Life Technologies). Two pulses were given at 2000 V. Following electroporation, cells were allowed to recover for 24 h at 22°C without selection antibiotics, after which they were plated onto hygromycin B rabbit blood agar plates. Colonies were picked, and Cas9-expressing cells were grown again in a liquid culture medium at 37°C. For gene deletion, the donor template (neomycin resistance cassette) and guide RNA were PCR amplified and purified using the GeneElute PCR purification kit (# HPPCRPKRO, Sigma). The donor template and guide RNA were electroporated into the Cas9-containing *L. major* using the same parameters as described previously. Following electroporation and recovery, cells were plated on G-418 containing rabbit blood agar plates. To identify the zygosity of the growing colonies, genomic DNA was extracted from individual colonies, and diagnostic PCR was performed. The PCR products were sequenced to confirm the developed KO. The KO strains were named N1, N2, N3 and N4. All primers used in the study are listed in Supplementary File S2.

### 2.5 Light microscopy

Cellular morphology was determined microscopically after staining with Giemsa stain (# GRM-945, HIMEDIA). Morphometric changes were observed using an EVOS inverted light microscope (# EVOS M7000, Life Technologies).

### 2.6 Transmission electron microscopy

WT and N2 KO strains of *Leishmania* were cultured to the log phase and fixed in glutaraldehyde fixative. The fixed cells were then embedded, sectioned, and stained as described previously with minor changes as described previously (Harvey et al., 2022; Schweer et al., 2023). These samples were viewed using a JEOL transmission electron microscope (TEM) (JEM-1400Plus) equipped with an XR81M-B Camera (Advanced Microscopy Techniques Corp) and camera software used was of version V602.

### 2.7 Peanut agglutination

Peanut agglutination (PNA) is a test that is used to separate procyclic and metacyclic promastigotes as the nonvirulent procyclic promastigotes have lectin content on their surface, which can be detected by PNA(Al-Bayati et al., 2017). *L. major* WT and KO strains at the stationary phase were resuspended in 1x PBS and incubated with peanut lectin (#L7381, Sigma) at a concentration of 100 μg/mL for 30 min at 25°C. The agglutinated parasites would settle down at the bottom of the tube and the metacyclic will float on the top of the PBS layer. Non-agglutinated parasites were counted using a hemocytometer and plotted as a percentage of the total parasite population.

### 2.8 Sample preparation for in-solution whole proteome analysis

WT and KO strains were harvested from the blood agar slant, washed three times with 1x PBS, and centrifuged to pellet the parasites. The cell pellet, containing a protease inhibitor cocktail (#11836170001, Roche), was dissolved in hot cell lysis buffer (6M guanidinium hydrochloride (#G4504, Sigma) and 0.1M Tris pH 8.5 (# 648310-M, Sigma) and mixed by vertexing. The mixture was then kept in a 90°C water bath for 10 min to ensure efficient cell lysis. To shear genomic DNA and reduce lysate viscosity, the mixture was sonicated at 40% amplitude for 1 min, followed by incubation at 90°C for 5 min. The lysate was then centrifuged at 15,000 g for 30 min at 4°C to pellet debris. Protein estimation was performed using the Bicinchoninic acid (BCA) protein estimation method (# A55860, Thermo Fisher). A total of 100 µg of protein was pelleted by chloroform (# 650498, Sigma) and methanol (# 179337, Sigma) protein precipitation. The protein pellet was dissolved in cell lysis buffer, followed by reduction and alkylation. Trypsin digestion was performed at a 1:20 trypsin (#T7575, Sigma) to protein ratio at 37°C overnight. Digestion was stopped by reducing the pH to less than three by adding formic acid (# 695076, Sigma). Desalting of the peptides was done using Waters C-18 Sep-Pak 1cc cartridges (# WAT023590, Waters).

### 2.9 Liquid chromatography-mass spectroscopy (LC-MS)

Dried peptides were reconstituted in 30 µL of 0.1% formic acid (# 28905, Thermofischer Scientific) and injected into the Nano LC/MS Orbitrap Eclipse™ Tribrid™ Mass Spectrometer (# FSN04-10003, Thermo Fisher). Peptides were separated on a 3-h gradient in a reverse phase column, ranging from 5% to 95% acetonitrile in 0.1% formic acid, with a flow rate of 300 nL/μl. MS scans (150–200 m/z) were performed at a resolution of 120,000 at 400 m/z and AGC of 400,000.

### 2.10 Mass spectroscopy data processing

Raw files from the spectroscopy runs were analyzed using MaxQuant version 1.6.1.4. Label-free quantification was performed with default parameters and enzyme trypsin. The raw data was searched against the *Leishmania* reference proteome UP000000542 available on UniProt. Statistical analysis of the MaxQuant output files was conducted using Perseus. KEGG Mapper and STRING were used for pathway analysis.

### 2.11 *In vivo* mice infection

To study the virulence of the WT and KO-developed strains, a parasite count of (10^6) was inoculated into the hind footpad of each mouse (five per group per time point). WT avirulent parasites were also included in the study as a positive control. Therefore there were total four groups: WT virulent, WT avirulent, N2, and N3. These mice were monitored for the development of skin lesions and infection over the period of 8 weeks post infection. Lesions were measured weekly post-inoculation using vernier calipers in all the groups of mice. Draining lymph nodes were isolated from mice at one, two-and 3 months post-infection and weighed by sacrificing five mice from each group per month.

### 2.12 Statistics

Statistical analysis was performed using Two Way Anova for growth curve analysis and foot pad thickness and One-way ANOVA was used for peanut agglutination and lymph node size measurements. GraphPad Prism (version 7.0) was used for plotting all the graphs.

## 3 Results

### 3.1 BLAST analysis

A PBLAST search in *L. major* against the Beta subunit of SNF1 kinase showed no homologous proteins (Supplementary Figure S1). However, a Gamma subunit PBLAST identified one Cystathionine β-Synthase (CBS) domain-containing protein (LMJF_35_0760) with a score of 55.1. Additionally, a PBLAST search against the *S*. *cerevisiae* SNF1 kinase alpha subunit identified five proteins in *L. major* with alignment scores above 200. Four of these five proteins contain the conserved STKc_AMPK_alpha domain. For convenience, the knockout strains of these proteins were named N1 (LMJF_36_0900), N2 (LMJF_29_0200), N3 (LMJF_18_0640), and N4 (LMJF_33_171), and this annotation was used throughout the paper. MSA of the selected genes with *S. cerevisiae* homologs (Supplementary Figure S1) showed a high degree of similarity in the N-terminal region containing the STKc_AMPK_alpha domain (first 300 amino acids) across all proteins, with little homology in the rest of the protein. Table 1 summarizes the PBLAST results.

**TABLE 1:** Proteins chosen for deletion studies and their BLAST score.

Protein Name/UniProt name	Blast score/e-value	Conserved domain
N1 LMJF_36_0900/Q4Q1Y6_LEIMA	323/1e-100	STKc_AMPK_alpha domain
N2 LMJF_29_2020/E9AE64_LEIMA	321/1e-99	STKc_AMPK_alpha domain
N3 LMJF_18_0640/Q4QDX7_LEIMA	234/8e-73	STKc_AMPK_alpha domain
N4 LMJF_33_1710/Q4Q416_LEIMA	210/8e-60	STKc_AMPK_alpha domain

### 3.2 Confirmation of KO and growth characteristics

**FIGURE 1:**
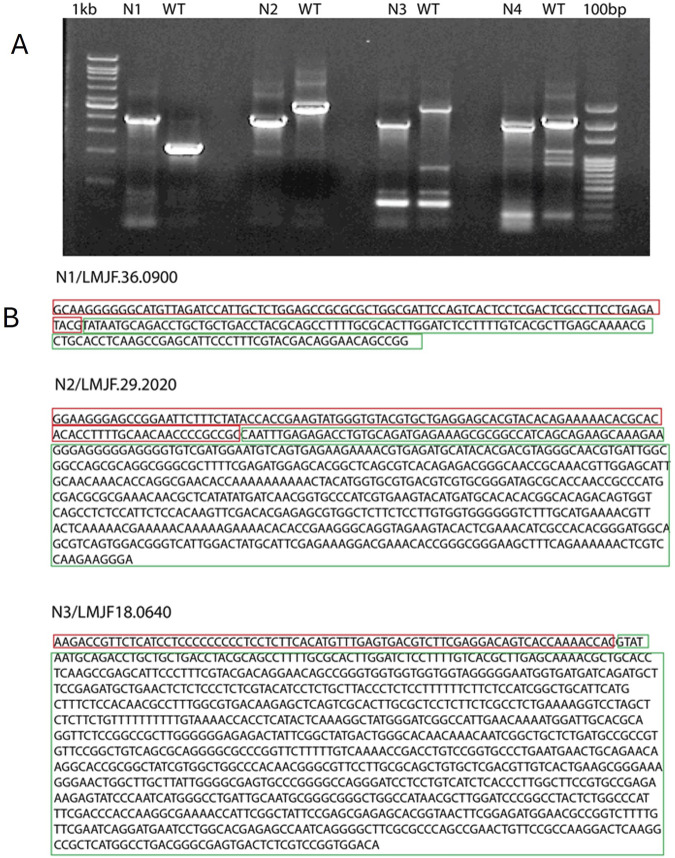
Confirmation of *Leishmania major* KOs and sequencing of the PCR products. **(A)** The PCR products of N1, N2, N3 were sequenced, the sequences marked in red correspond to WT DNA and those marked in green correspond to the inserted cassette. **(B)** Agarose gel electrophoresis of PCR products amplified from different KO strains. N1, N2 and N3 PCR show a single band of about 1800 base pairs which corresponds to the size of inserted cassette. N4 shows two bands, One corresponding to the WT gene fragment and the other to the inserted cassette.

After 1 week of electroporation with the Cas9 plasmid, *L. major* colonies appeared on blood agar plates. These colonies were picked and grown in G-418 supplemented RPMI1640 media or laid over blood agar slants, followed by DNA isolation. PCR reactions were set up using Open Reading Frame (ORF) flanking primers. A single band of the expected size (1750 base pairs) confirmed the generation of homozygous knockouts (KOs) (Figure 1A, lane 2,5 and 8). Two bands, one the size of the WT gene and the other the size of the inserted fragment, indicated a heterozygous KO (Figure 1A). The heterozygous KOs were further evaluated for zygosity after a second round of electroporation with a puromycin resistance cassette. The KO strains N1 (LMJF_36_0900), N2 (LMJF_29_0200), and N3 (LMJF_18_0640) were all achieved as homozygous KOs in the first round of selection with G-418. However, N4 (LMJF_33_171) was achieved as a heterozygous KO, containing two bands of the expected size (Figure 1B). Further electroporation with the puromycin resistance cassette followed by selection did not result in the development of a homozygous mutant, suggesting that this gene is essential. The KO strains N1, N2, and N3 with homozygous deletions were also validated by sequencing the PCR product, which confirmed the replacement of the target genomic DNA with the G-418 resistance cassette. Sequencing data also showed the joining of the 5′UTR of the gene. interest with the G-418 resistance cassette (Figure 1B). The N1 strain, showing similar characteristics to WT, was not studied for other experiments.

### 3.3 Growth characteristics

**FIGURE 2:**
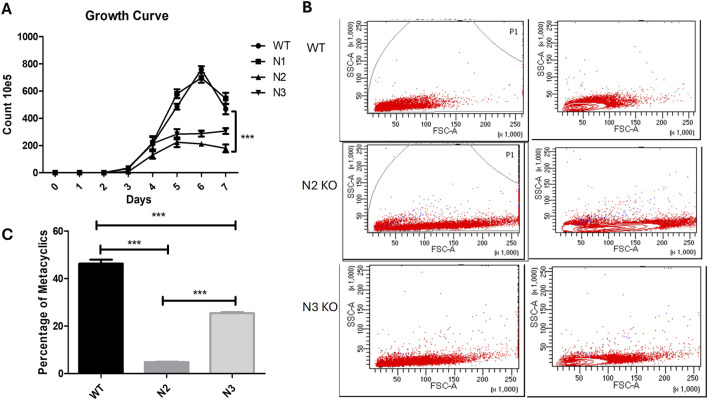
Characteristics of WT and KO strains and metacyclogenesis. **(A)** Growth curve analysis of WT, N1, N2 and N3 strains. Cells were seeded on blood agar slants overlaid with medium containing 10% serum at 1 × 10 ^5^ Cell/mL. Ten independent readings were taken at each time point for each sample. Results are obtained from three independent experiments with 10 readings captured in individual groups. Significant growth difference was found between WT vs N2 (***p < 0.001) and WT vs N3 (***p < 0.001) data is represented in mean ± SD, n = 10. **(B)** (Two Way Anova). Flowcytometry for WT, N2 and N3 KO strain showing variation in size and shape by increased forward scattered gated plot on the right side. N2 strain had showed increased FSC which described their bulbous shape. **(C)** Metacyclic parasite population quantification by peanut agglutination. Percentage of metacyclic parasite was significantly reduced in N2 strain when compared with WT (***p < 0.001) and WT vs. N3 (***p < 0.001) (One Way Anova). Data were represented by mean and SD value, n = 10.

Colonies of the four KO’s and the WT strain were picked and inoculated in liquid medium RPMI1640 supplemented with serum and appropriate selection antibiotic. Of the three homozygous KO’s, i.e., N1, N2, N3 only N1 was able to grow in liquid culture medium (RPMI1640 + 10%serum) in which WT cells grow, and the other strains N2 and N3 only grew in liquid medium when the culture medium was overlaid on rabbit blood agar slants. Heterozygous KO N4 was able to grow in liquid culture medium. A relative idea of the growth rate of *Leishmania* cells can be made by the time the first colonies appear on blood agar plates. WT colonies and N1 appear post 4–6 days of plating. However, the first colonies for the N2 and N3 strain appeared in the third- and second-week post-inoculation, respectively. For a generation of growth curve, all the strains were grown on blood agar slants overlaid with RPMI 1640 + 10%serum. The growth rate of the two strains N2 and N3 which were unable to grow in liquid culture medium without blood agar was much lower than that of WT strain (Figure 2A). These strains reached a much lower final cell density as compared to the WT, but highest cell density was reached only at 6^th^ or 7^th^ day in culture (***p < 0.001, WT vs. N2 and N3). WT and N1 strain showed similar growth pattern and after reaching the stationary phase there was a sharp decline in cell density of the WT and N1 culture (p > 0.05 WT vs. N1). Such sharp decline was not observed for N2 and the N3 strains (Figure 2A). Because of similar characteristics of WT and N1 strain, this strain was further not studied.

### 3.4 Altered cellular morphology in N2 KO strain

**FIGURE 3:**
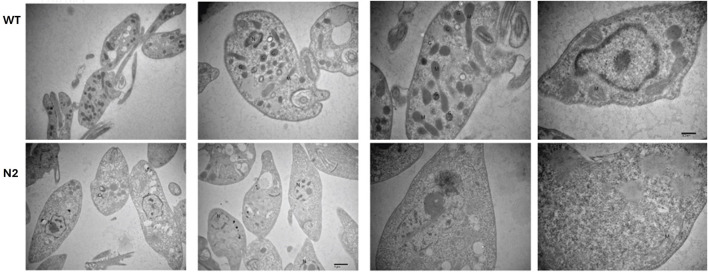
Transmission Electron Microscopy (TEM) of the WT and N2 strains. The WT micrograph shows numerous glycosomes and mitochondria distributed throughout the cellular cytoplasm. In contrast, the N2 strain electron micrograph reveals promastigotes lacking glycosomes and having fewer mitochondria. Additionally, the mitochondria in the N2 strain exhibit deformed, irregularly shaped cristae compared to those in the WT cells. N = nucleus, M = Mitochondria, G = Glycosomes.

The cellular morphology of the KO strains were confirmed by flow cytometry. WT strain showed reduced FSC area which indicated smaller size of the parasite (Figure 2B, Top gated panel). N2 strain cells became bulbous and enlarged, with shortened flagella which showed increased FSC gated cell population (Figure 2B 2^nd^ lower gated panel). Similarly, the N3 strain showed increased FSC as compared to WT cells (Figures 2B, 3^rd^ lower gated panel).

### 3.5 Non-agglutination property of N2 and N3 KO strains

Metacyclogenesis was found to be reduced in the N2 and N3 strain stationary phases compared to the WT virulent strain. The WT virulent strain at the stationary phase had a 46.2% ± 5.3% metacyclic parasite population. This percentage was reduced to 4.8% ± 0.52% and 25.4% ± 1.27% in the N2 and N3 strains, respectively (***p < 0.001) (Figure 2C). Peanut lectin did not bind to metacyclic parasites and only agglutinates procyclic parasites, leaving only virulent metacyclic parasites in suspension.

### 3.6 Ultrastructural changes in KO N2 strain

TEM was performed to identify ultrastructural alterations in the WT and N2 strains. As N2 and N3 strains resulted in similar patterns of growth characteristics, only the N2 strain was studied for the ultrastructural changes. Figure 3 projects, representative ultrastructural images of WT (upper panel) and N2 KO (lower panel) promastigotes. The N2 strain (Figure 3 lower panel) was found to be completely devoid of glycosomes, whereas WT *Leishmania* promastigotes typically contain 10–20 glycosomes per cell (Figure 3 upper panel). Additionally, the mitochondria in the N2 strain appeared deformed with irregular cristae which were fewer in number compared to the WT strain (Figure 3). The health of mitochondria is known to be controlled by the SNF1 kinase, indicating a significant impact on the N2 strain’s cellular structure.

### 3.7 Proteomics analysis

**FIGURE 4:**
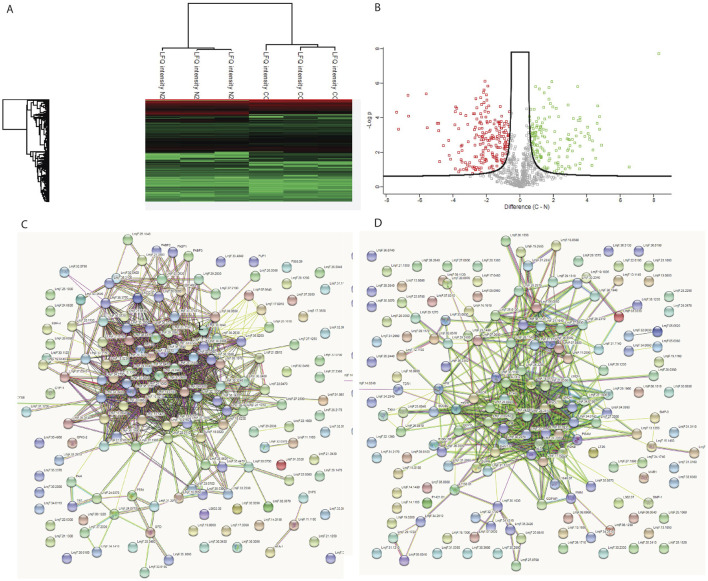
Whole proteome analysis of WT and N2 strains. **(A)** Heat map showing the differentially expressed proteins between the two strains. **(B)** Volcano plot illustrates the distribution of differentially expressed proteins, with green dots representing proteins with increased expression and red dots representing proteins with decreased expression in the N2 strain. **(C)** STRING pathway analysis of upregulated proteins in the WT. **(D)** STING pathway analysis for N2 strain. The clustering of proteins in the STRING pathway indicates that upregulated proteins are part of specific pathways and not randomly distributed, suggesting that the KO affects entire pathways rather than individual proteins at random.

As N2 and N3 strains resulted in similar patterns of growth characteristics, only N2 strain was studied for the whole proteomics. To investigate the underlying basis of the changes observed in the N2 strain, a whole proteome analysis was performed. The WT and N2 were grown on blood agar slants in culture medium. After harvesting, cells were processed for mass spectrometry. The raw data obtained from mass spectrometry were processed using MaxQuant, identifying a total of 2,227 different proteins (Supplementary File S2). Perseus software was used for data analysis and graphical representation. Out of the 2,227 proteins, 429 were found to be differentially expressed at FDR = 0.05 and s0 = 0.4. Among these, 207 proteins were upregulated and 222 were downregulated. KEGG mapper was used to categorize these proteins into functional categories and pathways (Figure 4). Overall, metabolism was found to be downregulated in the N2 strain, with major pathways affected including glycolysis/gluconeogenesis, biosynthesis and degradation of amino acids, pyruvate metabolism and the citric acid cycle, fatty acid metabolism, and fatty acid synthesis and degradation (Supplementary File S3). There was also decreased expression of ROS and NOS scavenging enzymes located in glycosomes. A fraction of upregulated proteins belonged to the translation machinery, including various ribosomal proteins, proteins involved in ribosome biogenesis, and tRNA transport. The mass spectrometry proteomics data have been deposited to the ProteomeXchange Consortium *via* the PRIDE partner repository with the dataset identifier PXD028966.

### 3.8 N2 KO strain failed to develop cutaneous leishmaniasis

**FIGURE 5:**
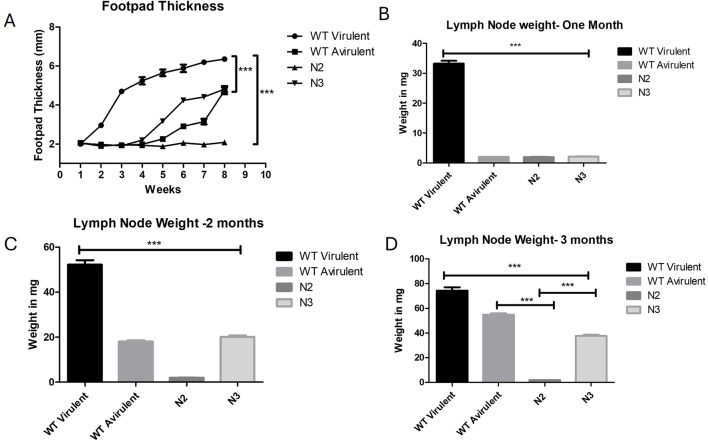
Loss of virulence in N2 KO strain and development of infection in mice. **(A)** Foot pad thickness was measured for 8 weeks, per week post infection. No foot pad swelling was observed in the N2 infected mice. Five mice were taken for each time point and infection was given in both the hind foot pads (n = 10) ***, P < 0.001 (Two Way Anova). **(B)** Draining lymph node weight was measured at 1 month, **(C)** at 2 months post infection and **(D)** at 3 months post infection. Significantly increased lymph node weight was observed in WT mice in all 3 months post infection when compared with other groups (***p < 0.001). N2 strain infected mice. lymph node showed unaltered weight over the period of 1 to 3 months and remained constant when compared with WT mice (***p < 0.001). At 3 months post infection, WT virulent and N3 mice showed increased lymph node weight significantly increased when compared to N2 mice (***p < 0.001). Data are represented by mean ± SD, n = 10 (One Way Anova).

In order to study the virulence of KO strains in mice, N2, N3 KO promastigotes infection was induced by inoculating mice hind footpads with stationary phase parasites. WT virulent and WT avirulent parasites were also inoculated in mice’s hind foot pads as a positive control. Footpad thickness was found to be gradually increased in WT virulent mice and showed a maximum of 6.35 mm lymph node size at week eight post infection when compared to WT avirulent with a mean of 4.7 mm (***p < 0.001), N2 with a mean of 2.0 mm (***p < 0.001), N3 with a mean of 4.8 mm (***p < 0.001) (Figure 5A). Representative images of footpad lesions from different groups of mice for 1 month post infection are described in Supplementary Figure S1 and for 2 months in Supplementary Figure S2 and Supplementary Figure S3.

Lymph node was also extracted from all group of mice at 1,2- and 3-month post infection and the weighted to corelate the parasite burden. At 1 month post infection the highest lymph node weight was found in the WT virulent mice when compared to all other groups (***p < 0.001) (Figure 5B). Representative images of lymph node for 1 month post infection are shown in Supplementary Figure S4. The lymph node size was found to be gradually increasing at 2 and 3 months post infection in mice infected with avirulent WT parasites and N3 strain but still was significantly lower when compared to WT virulent mice (***p < 0.001) (Figures 5C, D). The lymph node weight of the N2-infected mice did not increase till 3 months post-infection when compared with WT virulent or WT avirulent mice (N2 vs. WT avirulent***p < 0.001, N2 vs. N3 ***p < 0.001) Figure 5D. Lymph node representative images for 2 and 3 months are showed in Supplementary Figure S5 and Supplementary Figure s6. These results suggest that N2 strain failed to develop any lesions in mice whereas N3 KO regained the virulence over the period of time.

The infection caused by the N3 strain resulted in reduced lesion size and lymph node weight. The WT avirulent strain was used as a reference for a less virulent strain. The disease caused by this strain was delayed by 4 weeks in terms of lesion appearance, lesion growth, and lymph node weight. Although the avirulent strain eventually reached the same lesion size and lymph node weight as the virulent strain, it was delayed by 4 weeks. In contrast, the WT virulent strain caused severe disease, characterized by footpad and lymph node swelling, accompanied by an increase in lymph node weight. These results indicate that N2 KO strain has failed to develop any infectious symptoms in mice and lost complete virulence.

## 4 Discussion

Parasitic infections are one of the most complex microorganisms that are difficult to treat. Vaccine development is difficult because of the complex life cycle in multiple invertebrate and vertebrate hosts (Cummings et al., 2022). Many natural plant extracts, natural drugs, or conventional drugs have been studied against protozoan parasites like *Plasmodium*, *Leishmania* and *Toxoplasma* (Anand et al., 2015; Anand et al., 2016; Joachim, 2016). *Leishmania* is a protozoan parasite causing visceral, cutaneous, and mucocutaneous leishmaniasis worldwide, and many treatment strategies, such as natural and synthetic compounds, are shown to be effective against this parasite (Kaur, Chauhan et al. , Anand, 2024; Chauhan et al., 2024). Vaccines are under the experimental stage against leishmaniasis because of its complex life cycle and the conventional available drug amphotericin B is the most potent drug known but has toxicity and cost-effectiveness (Shirzadi, 2019). Therefore, in the present study, we tried to identify vaccine targets for leishmaniasis, which could be further used in large-scale studies.

We characterized a homolog of the *S. cerevisiae* SNF1/AMPK catalytic domain alpha subunit in *L. major*. We successfully developed three homozygous gene KO’s named N1, N2, and N3. *L. major* parasite is known to reach a higher cell density on rabbit blood agar slants because of its nutritional composition as compared to HIFBS-supplemented media. Also, the differentiation from amastigote to promastigote form is achieved faster on blood agar (Steiger and Steiger, 1977; Ullah et al., 2018). Therefore, nutritional requirements were evaluated by growing the strains on blood agar and then transferring them to a liquid culture medium with HIFBS. N2 and N3 strains died in liquid culture but were found to be grown on blood agar plates and liquid culture overlaid on rabbit blood agar slants. Cell clumping, observed in low-passage *L. major* strains, is lost with increased passages and correlates with loss of virulence. The N1 and heterozygote N4 strains showed characteristic cell clumping and no differential growth requirements, so they were not chosen for virulence studies.

This suggests essential nutrients in blood agar are required for their growth. WT cells behaved to bypass this need by switching to alternate pathways, but the SNF1 kinase mutants are unable to switch, similar to yeast SNF1 kinase mutants. N2 strain cells become bulbous with shortened flagella and increased cell size, showing slower growth at the stationary phase. High cell density is stressful for microorganisms, and various mechanisms help them survive nutrient-limiting conditions. *Leishmania* undergoes metacyclogenesis at the stationary phase, producing metacyclic parasites that enter human macrophages and cause disease (Serafim et al., 2012). Peanut butter agglutination shows a reduction of metacyclic in the N2 strain at the stationary phase, highlighting the role of SNF1 kinase in stress adaptation and metacyclogenesis.

SNF1 kinase in *S. cerevisiae* controls peroxisome proliferation, and yeast null mutants lack peroxisomes (Simon et al., 1992). *Leishmania* has related organelles called glycosomes, which contain peroxisomal and glycolytic enzymes (Jamdhade et al., 2015). Our electron micrograph data shows the absence of glycosomes in the N2 strain of *L. major* promastigotes. The proteome analysis also indicates decreased expression of peroxisomal enzymes which act as ROS scavengers. Mitochondria, another organelle regulated by SNF1 kinase in *S. cerevisiae* (Zhang et al., 2018), are essential for respiration during glucose starvation. Our study shows a reduction in the number of mitochondria and alterations in mitochondrial cristae structure in the N2 strain. These changes are reflected in the proteome data, which shows reduced expression of most metabolic enzymes in *L. major*. This suggests that SNF1 kinase is crucial for maintaining glycosomes and mitochondrial health in *Leishmania.*

In our whole proteome study, significant proteome change from a single gene deletion highlights the KO N2 strain role in energy metabolism and stress adaptation in *L. major*. KEGG pathway analysis revealed the highest number of upregulated proteins in ribosomes, with many ribosomal and tRNA binding proteins upregulated in the N2 strain. Increased ribosomal protein expression, typically linked to cellular growth, also suggests a role in stress adaptation, as seen in rice plants under dehydration stress. Transgenic rice having over-expression of the ribosomal proteins RPL23A, was found to be better adapted to drought and salt stress (Moin et al., 2017). However, this increase is not universal across all stress types. For instance, orbiculata1 (orb1) mutants with defective glutamate synthase also showed increased ribosomal protein expression, correlating with reduced growth (Munoz-Nortes et al., 2017). The N2 strain exhibited a general decrease in metabolic processes, with reduced expression of enzymes involved in carbohydrate, lipid, and amino acid metabolism, as well as peroxisomal enzymes for ROS and NOS scavenging. Proteome data aligned well with electron microscopy findings, showing N2 KO parasite with lack of glycosomes, deformed mitochondria, and downregulated metabolism. Role of N2 KO can be further studied to study the innate immune response in mice and also to study the role of effector molecules as studied previously in other protozoan parasite (Lutshumba et al., 2020; Anand et al., 2022).

*In vivo* mice experiment with the N2 strain resulted in no footpad lesions or lymph node enlargement after 3 months post infection. WT virulent infected mice showed increased footpad lesion and lymph node weight over the period of time. At 2 months post treatment, N3 strain and even avirulent stain of mice showed increased food pad lesion size and lymph node enlargement. At 3 months post infection, except N2 infected mice, all other groups showed increased footpad lesion and lymph node size increase. This loss of virulence may be due to fewer metacyclics in N2, which are crucial for surviving immune complement lysis and establishing disease in host macrophages. The N2 strain stringent nutritional requirements and slower growth rate may also contribute to its loss of virulence in mice model.

## 5 Conclusion

The SNF1 kinase enzymatic alpha subunit plays a crucial role in stress adaptation and energy homeostasis in *Leishmania*. Its loss leads to altered metabolism and reduced metacyclogenesis, rendering the N2 strain avirulent in mouse models. This underscores the importance of this protein in drug discovery and the development of live attenuated strains in *Leishmania*.

## Data Availability

The datasets presented in this study can be found in online repositories. The names of the repository/repositories and accession number(s) can be found in the article/Supplementary Material.
