# Supplementary figures and images for: Identification and characterization of yeast SNF1 kinase homologs in Leishmania major
Source: Front Mol Biosci. 2025 Mar 24;12:1567703. doi: 10.3389/fmolb.2025.1567703 (PMC11973601; doi:10.3389/fmolb.2025.1567703)

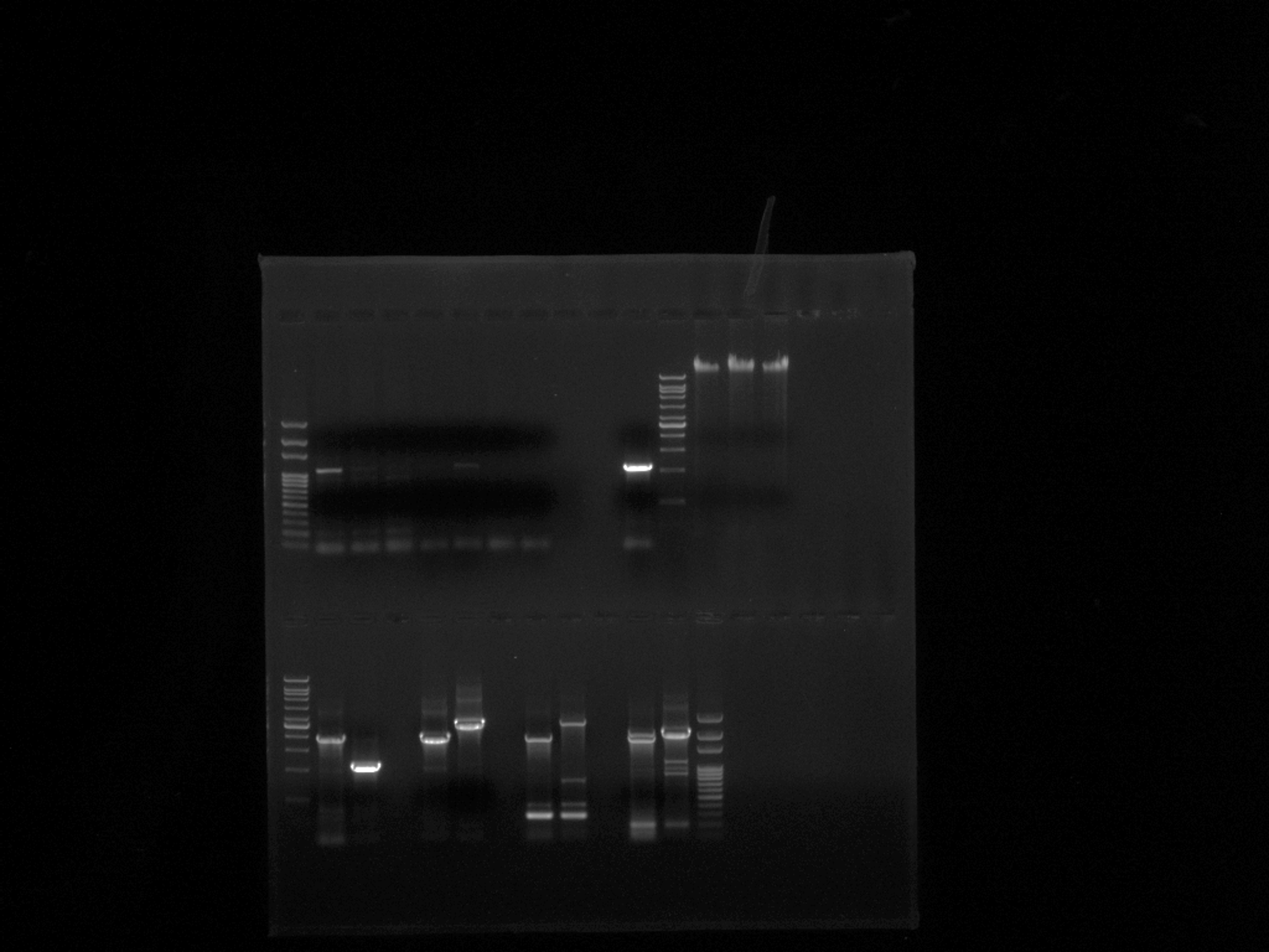

Supplement: Supplementary file 2 [file Image1.tif]
