# Supplementary material for: Identification and characterization of yeast SNF1 kinase homologs in Leishmania major
Source: Front Mol Biosci. 2025 Mar 24;12:1567703. doi: 10.3389/fmolb.2025.1567703 (PMC11973601; doi:10.3389/fmolb.2025.1567703)
Supplement: Supplementary file 3 [file Supplementaryfile2.docx]

**Identification and characterization of yeast SNF1 kinase homologs in *Leishmania major***

Gaurav Shoeran^1,#^, Namrata Anand^1,$^, Upninder kaur^1^ Kapil Goyal^4^ and Rakesh Sehgal^1^*

1. Department of Medical Parasitology, Postgraduate Institute of Medical Education and Research, Chandigarh, India
2. Department of Virology, Postgraduate Institute of Medical Education and Research, Chandigarh, India.

**Current Address:**

# Department of Pharmacy Practice and Science, College of Pharmacy, University of Kentucky, Ky, USA.

$ Department of Hematology and Oncology, College of Medicine, University of Chicago, IL, USA.

* Aarupudai Veedu Medical College & Hospital, Puducherry, India

***Corresponding author:**

Dr. Rakesh Sehgal, Dean, and Professor of Microbiology, Aarupudai Veedu Medical College & Hospital, Puducherry, India

Email- [sehgalpgi@gmail.com](mailto:sehgalpgi@gmail.com)

**Supplementary data file 2**

**Pathways upregulated in the N2 KO**

[lma03010](https://www.genome.jp/kegg-bin/show_pathway?161298516583721/lma03010.args) Ribosome - Leishmania major ([60](javascript:display('lma03010')))

[lma01100](https://www.genome.jp/kegg-bin/show_pathway?161298516583721/lma01100.args) Metabolic pathways - Leishmania major ([20](javascript:display('lma01100')))

[lma01110](https://www.genome.jp/kegg-bin/show_pathway?161298516583721/lma01110.args) Biosynthesis of secondary metabolites - Leishmania major ([11](javascript:display('lma01110')))

[lma03013](https://www.genome.jp/kegg-bin/show_pathway?161298516583721/lma03013.args) RNA transport - Leishmania major ([10](javascript:display('lma03013')))

[lma01240](https://www.genome.jp/kegg-bin/show_pathway?161298516583721/lma01240.args) Biosynthesis of cofactors - Leishmania major ([6](javascript:display('lma01240')))

[lma03008](https://www.genome.jp/kegg-bin/show_pathway?161298516583721/lma03008.args) Ribosome biogenesis in eukaryotes - Leishmania major ([6](javascript:display('lma03008')))

[lma00970](https://www.genome.jp/kegg-bin/show_pathway?161298516583721/lma00970.args) Aminoacyl-tRNA biosynthesis - Leishmania major ([6](javascript:display('lma00970')))

[lma01230](https://www.genome.jp/kegg-bin/show_pathway?161298516583721/lma01230.args) Biosynthesis of amino acids - Leishmania major ([5](javascript:display('lma01230')))

[lma00270](https://www.genome.jp/kegg-bin/show_pathway?161298516583721/lma00270.args) Cysteine and methionine metabolism - Leishmania major ([4](javascript:display('lma00270')))

[lma00230](https://www.genome.jp/kegg-bin/show_pathway?161298516583721/lma00230.args) Purine metabolism - Leishmania major ([4](javascript:display('lma00230')))

[lma03018](https://www.genome.jp/kegg-bin/show_pathway?161298516583721/lma03018.args) RNA degradation - Leishmania major ([3](javascript:display('lma03018')))

[lma00620](https://www.genome.jp/kegg-bin/show_pathway?161298516583721/lma00620.args) Pyruvate metabolism - Leishmania major ([3](javascript:display('lma00620')))

[lma00360](https://www.genome.jp/kegg-bin/show_pathway?161298516583721/lma00360.args) Phenylalanine metabolism - Leishmania major ([3](javascript:display('lma00360')))

[lma00400](https://www.genome.jp/kegg-bin/show_pathway?161298516583721/lma00400.args) Phenylalanine, tyrosine and tryptophan biosynthesis - Leishmania major ([3](javascript:display('lma00400')))

[lma01200](https://www.genome.jp/kegg-bin/show_pathway?161298516583721/lma01200.args) Carbon metabolism - Leishmania major ([3](javascript:display('lma01200')))

[lma00061](https://www.genome.jp/kegg-bin/show_pathway?161298516583721/lma00061.args) Fatty acid biosynthesis - Leishmania major ([2](javascript:display('lma00061')))

[lma00350](https://www.genome.jp/kegg-bin/show_pathway?161298516583721/lma00350.args) Tyrosine metabolism - Leishmania major ([2](javascript:display('lma00350')))

[lma00790](https://www.genome.jp/kegg-bin/show_pathway?161298516583721/lma00790.args) Folate biosynthesis - Leishmania major ([2](javascript:display('lma00790')))

[lma03040](https://www.genome.jp/kegg-bin/show_pathway?161298516583721/lma03040.args) Spliceosome - Leishmania major ([2](javascript:display('lma03040')))

[lma00730](https://www.genome.jp/kegg-bin/show_pathway?161298516583721/lma00730.args) Thiamine metabolism - Leishmania major ([2](javascript:display('lma00730')))

[lma04141](https://www.genome.jp/kegg-bin/show_pathway?161298516583721/lma04141.args) Protein processing in endoplasmic reticulum - Leishmania major ([2](javascript:display('lma04141')))

[lma01212](https://www.genome.jp/kegg-bin/show_pathway?161298516583721/lma01212.args) Fatty acid metabolism - Leishmania major ([2](javascript:display('lma01212')))

[lma03030](https://www.genome.jp/kegg-bin/show_pathway?161298516583721/lma03030.args) DNA replication - Leishmania major ([1](javascript:display('lma03030')))

[lma00100](https://www.genome.jp/kegg-bin/show_pathway?161298516583721/lma00100.args) Steroid biosynthesis - Leishmania major ([1](javascript:display('lma00100')))

[lma00030](https://www.genome.jp/kegg-bin/show_pathway?161298516583721/lma00030.args) Pentose phosphate pathway - Leishmania major ([1](javascript:display('lma00030')))

[lma01210](https://www.genome.jp/kegg-bin/show_pathway?161298516583721/lma01210.args) 2-Oxocarboxylic acid metabolism - Leishmania major ([1](javascript:display('lma01210')))

[lma00561](https://www.genome.jp/kegg-bin/show_pathway?161298516583721/lma00561.args) Glycerolipid metabolism - Leishmania major ([1](javascript:display('lma00561')))

[lma04146](https://www.genome.jp/kegg-bin/show_pathway?161298516583721/lma04146.args) Peroxisome - Leishmania major ([1](javascript:display('lma04146')))

[lma00240](https://www.genome.jp/kegg-bin/show_pathway?161298516583721/lma00240.args) Pyrimidine metabolism - Leishmania major ([1](javascript:display('lma00240')))

[lma00330](https://www.genome.jp/kegg-bin/show_pathway?161298516583721/lma00330.args) Arginine and proline metabolism - Leishmania major ([1](javascript:display('lma00330')))

[lma00260](https://www.genome.jp/kegg-bin/show_pathway?161298516583721/lma00260.args) Glycine, serine and threonine metabolism - Leishmania major ([1](javascript:display('lma00260')))

[lma00250](https://www.genome.jp/kegg-bin/show_pathway?161298516583721/lma00250.args) Alanine, aspartate and glutamate metabolism - Leishmania major ([1](javascript:display('lma00250')))

[lma03060](https://www.genome.jp/kegg-bin/show_pathway?161298516583721/lma03060.args) Protein export - Leishmania major ([1](javascript:display('lma03060')))

[lma03430](https://www.genome.jp/kegg-bin/show_pathway?161298516583721/lma03430.args) Mismatch repair - Leishmania major ([1](javascript:display('lma03430')))

[lma00860](https://www.genome.jp/kegg-bin/show_pathway?161298516583721/lma00860.args) Porphyrin and chlorophyll metabolism - Leishmania major ([1](javascript:display('lma00860')))

[lma03420](https://www.genome.jp/kegg-bin/show_pathway?161298516583721/lma03420.args) Nucleotide excision repair - Leishmania major ([1](javascript:display('lma03420')))

[lma05140](https://www.genome.jp/kegg-bin/show_pathway?161298516583721/lma05140.args) Leishmaniasis - Leishmania major ([1](javascript:display('lma05140')))

[lma00190](https://www.genome.jp/kegg-bin/show_pathway?161298516583721/lma00190.args) Oxidative phosphorylation - Leishmania major ([1](javascript:display('lma00190')))

[lma00630](https://www.genome.jp/kegg-bin/show_pathway?161298516583721/lma00630.args) Glyoxylate and dicarboxylate metabolism - Leishmania major ([1](javascript:display('lma00630')))

[lma00071](https://www.genome.jp/kegg-bin/show_pathway?161298516583721/lma00071.args) Fatty acid degradation - Leishmania major ([1](javascript:display('lma00071')))

[lma03020](https://www.genome.jp/kegg-bin/show_pathway?161298516583721/lma03020.args) RNA polymerase - Leishmania major ([1](javascript:display('lma03020')))

[lma00640](https://www.genome.jp/kegg-bin/show_pathway?161298516583721/lma00640.args) Propanoate metabolism - Leishmania major ([1](javascript:display('lma00640')))

[lma00220](https://www.genome.jp/kegg-bin/show_pathway?161298516583721/lma00220.args) Arginine biosynthesis - Leishmania major ([1](javascript:display('lma00220')))

[lma03015](https://www.genome.jp/kegg-bin/show_pathway?161298516583721/lma03015.args) mRNA surveillance pathway - Leishmania major ([1](javascript:display('lma03015')))

[lma00564](https://www.genome.jp/kegg-bin/show_pathway?161298516583721/lma00564.args) Glycerophospholipid metabolism - Leishmania major ([1](javascript:display('lma00564')))

[lma00450](https://www.genome.jp/kegg-bin/show_pathway?161298516583721/lma00450.args) Selenocompound metabolism - Leishmania major ([1](javascript:display('lma00450')))

[lma00130](https://www.genome.jp/kegg-bin/show_pathway?161298516583721/lma00130.args) Ubiquinone and other terpenoid-quinone biosynthesis - Leishmania major ([1](javascript:display('lma00130')))

**Pathways downregulated in the N2 KO**

[lma_M00009](https://www.genome.jp/kegg-bin/show_module?161298619873191/lma_M00009.args) Citrate cycle (TCA cycle, Krebs cycle) ([7](javascript:display('lma_M00009')))

[lma_M00165](https://www.genome.jp/kegg-bin/show_module?161298619873191/lma_M00165.args) Reductive pentose phosphate cycle (Calvin cycle) ([6](javascript:display('lma_M00165')))

[lma_M00003](https://www.genome.jp/kegg-bin/show_module?161298619873191/lma_M00003.args) Gluconeogenesis, oxaloacetate => fructose-6P ([6](javascript:display('lma_M00003')))

[lma_M00001](https://www.genome.jp/kegg-bin/show_module?161298619873191/lma_M00001.args) Glycolysis (Embden-Meyerhof pathway), glucose => pyruvate ([6](javascript:display('lma_M00001')))

[lma_M00012](https://www.genome.jp/kegg-bin/show_module?161298619873191/lma_M00012.args) Glyoxylate cycle ([5](javascript:display('lma_M00012')))

[lma_M00002](https://www.genome.jp/kegg-bin/show_module?161298619873191/lma_M00002.args) Glycolysis, core module involving three-carbon compounds ([5](javascript:display('lma_M00002')))

[lma_M00011](https://www.genome.jp/kegg-bin/show_module?161298619873191/lma_M00011.args) Citrate cycle, second carbon oxidation, 2-oxoglutarate => oxaloacetate ([5](javascript:display('lma_M00011')))

[lma_M00168](https://www.genome.jp/kegg-bin/show_module?161298619873191/lma_M00168.args) CAM (Crassulacean acid metabolism), dark ([4](javascript:display('lma_M00168')))

[lma_M00167](https://www.genome.jp/kegg-bin/show_module?161298619873191/lma_M00167.args) Reductive pentose phosphate cycle, glyceraldehyde-3P => ribulose-5P ([4](javascript:display('lma_M00167')))

[lma_M00171](https://www.genome.jp/kegg-bin/show_module?161298619873191/lma_M00171.args) C4-dicarboxylic acid cycle, NAD - malic enzyme type ([4](javascript:display('lma_M00171')))

[lma_M00036](https://www.genome.jp/kegg-bin/show_module?161298619873191/lma_M00036.args) Leucine degradation, leucine => acetoacetate + acetyl-CoA ([3](javascript:display('lma_M00036')))

[lma_M00087](https://www.genome.jp/kegg-bin/show_module?161298619873191/lma_M00087.args) beta-Oxidation ([3](javascript:display('lma_M00087')))

[lma_M00050](https://www.genome.jp/kegg-bin/show_module?161298619873191/lma_M00050.args) Guanine ribonucleotide biosynthesis IMP => GDP,GTP ([2](javascript:display('lma_M00050')))

[lma_M00053](https://www.genome.jp/kegg-bin/show_module?161298619873191/lma_M00053.args) Pyrimidine deoxyribonuleotide biosynthesis, CDP/CTP => dCDP/dCTP,dTDP/dTTP ([2](javascript:display('lma_M00053')))

[lma_M00049](https://www.genome.jp/kegg-bin/show_module?161298619873191/lma_M00049.args) Adenine ribonucleotide biosynthesis, IMP => ADP,ATP ([2](javascript:display('lma_M00049')))

[lma_M00740](https://www.genome.jp/kegg-bin/show_module?161298619873191/lma_M00740.args) Methylaspartate cycle ([2](javascript:display('lma_M00740')))

[lma_M00004](https://www.genome.jp/kegg-bin/show_module?161298619873191/lma_M00004.args) Pentose phosphate pathway (Pentose phosphate cycle) ([2](javascript:display('lma_M00004')))

[lma_M00166](https://www.genome.jp/kegg-bin/show_module?161298619873191/lma_M00166.args) Reductive pentose phosphate cycle, ribulose-5P => glyceraldehyde-3P ([2](javascript:display('lma_M00166')))

[lma_M00010](https://www.genome.jp/kegg-bin/show_module?161298619873191/lma_M00010.args) Citrate cycle, first carbon oxidation, oxaloacetate => 2-oxoglutarate ([2](javascript:display('lma_M00010')))

[lma_M00552](https://www.genome.jp/kegg-bin/show_module?161298619873191/lma_M00552.args) D-galactonate degradation, De Ley-Doudoroff pathway, D-galactonate => glycerate-3P ([2](javascript:display('lma_M00552')))

[lma_M00173](https://www.genome.jp/kegg-bin/show_module?161298619873191/lma_M00173.args) Reductive citrate cycle (Arnon-Buchanan cycle) ([2](javascript:display('lma_M00173')))

[lma_M00085](https://www.genome.jp/kegg-bin/show_module?161298619873191/lma_M00085.args) Fatty acid elongation in mitochondria ([2](javascript:display('lma_M00085')))

[lma_M00308](https://www.genome.jp/kegg-bin/show_module?161298619873191/lma_M00308.args) Semi-phosphorylative Entner-Doudoroff pathway, gluconate => glycerate-3P ([2](javascript:display('lma_M00308')))

[lma_M00032](https://www.genome.jp/kegg-bin/show_module?161298619873191/lma_M00032.args) Lysine degradation, lysine => saccharopine => acetoacetyl-CoA ([2](javascript:display('lma_M00032')))

[lma_M00114](https://www.genome.jp/kegg-bin/show_module?161298619873191/lma_M00114.args) Ascorbate biosynthesis, plants, fructose-6P => ascorbate ([2](javascript:display('lma_M00114')))

[lma_M00019](https://www.genome.jp/kegg-bin/show_module?161298619873191/lma_M00019.args) Valine/isoleucine biosynthesis, pyruvate => valine / 2-oxobutanoate => isoleucine ([1](javascript:display('lma_M00019')))

[lma_M00376](https://www.genome.jp/kegg-bin/show_module?161298619873191/lma_M00376.args) 3-Hydroxypropionate bi-cycle ([1](javascript:display('lma_M00376')))

[lma_M00006](https://www.genome.jp/kegg-bin/show_module?161298619873191/lma_M00006.args) Pentose phosphate pathway, oxidative phase, glucose 6P => ribulose 5P ([1](javascript:display('lma_M00006')))

[lma_M00632](https://www.genome.jp/kegg-bin/show_module?161298619873191/lma_M00632.args) Galactose degradation, Leloir pathway, galactose => alpha-D-glucose-1P ([1](javascript:display('lma_M00632')))

[lma_M00013](https://www.genome.jp/kegg-bin/show_module?161298619873191/lma_M00013.args) Malonate semialdehyde pathway, propanoyl-CoA => acetyl-CoA ([1](javascript:display('lma_M00013')))

[lma_M00170](https://www.genome.jp/kegg-bin/show_module?161298619873191/lma_M00170.args) C4-dicarboxylic acid cycle, phosphoenolpyruvate carboxykinase type ([1](javascript:display('lma_M00170')))

[lma_M00141](https://www.genome.jp/kegg-bin/show_module?161298619873191/lma_M00141.args) C1-unit interconversion, eukaryotes ([1](javascript:display('lma_M00141')))

[lma_M00119](https://www.genome.jp/kegg-bin/show_module?161298619873191/lma_M00119.args) Pantothenate biosynthesis, valine/L-aspartate => pantothenate ([1](javascript:display('lma_M00119')))

[lma_M00845](https://www.genome.jp/kegg-bin/show_module?161298619873191/lma_M00845.args) Arginine biosynthesis, glutamate => acetylcitrulline => arginine ([1](javascript:display('lma_M00845')))

[lma_M00375](https://www.genome.jp/kegg-bin/show_module?161298619873191/lma_M00375.args) Hydroxypropionate-hydroxybutylate cycle ([1](javascript:display('lma_M00375')))

[lma_M00844](https://www.genome.jp/kegg-bin/show_module?161298619873191/lma_M00844.args) Arginine biosynthesis, ornithine => arginine ([1](javascript:display('lma_M00844')))

[lma_M00007](https://www.genome.jp/kegg-bin/show_module?161298619873191/lma_M00007.args) Pentose phosphate pathway, non-oxidative phase, fructose 6P => ribose 5P ([1](javascript:display('lma_M00007')))

[lma_M00098](https://www.genome.jp/kegg-bin/show_module?161298619873191/lma_M00098.args) Acylglycerol degradation ([1](javascript:display('lma_M00098')))

[lma_M00021](https://www.genome.jp/kegg-bin/show_module?161298619873191/lma_M00021.args) Cysteine biosynthesis, serine => cysteine ([1](javascript:display('lma_M00021')))

[lma_M00532](https://www.genome.jp/kegg-bin/show_module?161298619873191/lma_M00532.args) Photorespiration ([1](javascript:display('lma_M00532')))

[lma_M00128](https://www.genome.jp/kegg-bin/show_module?161298619873191/lma_M00128.args) Ubiquinone biosynthesis, eukaryotes, 4-hydroxybenzoate + polyprenyl-PP => ubiquinol ([1](javascript:display('lma_M00128')))

[lma_M00570](https://www.genome.jp/kegg-bin/show_module?161298619873191/lma_M00570.args) Isoleucine biosynthesis, threonine => 2-oxobutanoate => isoleucine ([1](javascript:display('lma_M00570')))

[lma_M00373](https://www.genome.jp/kegg-bin/show_module?161298619873191/lma_M00373.args) Ethylmalonyl pathway ([1](javascript:display('lma_M00373')))

[lma_M00029](https://www.genome.jp/kegg-bin/show_module?161298619873191/lma_M00029.args) Urea cycle ([1](javascript:display('lma_M00029')))

[lma_M00052](https://www.genome.jp/kegg-bin/show_module?161298619873191/lma_M00052.args) Pyrimidine ribonucleotide biosynthesis, UMP => UDP/UTP,CDP/CTP ([1](javascript:display('lma_M00052')))

[lma_M00741](https://www.genome.jp/kegg-bin/show_module?161298619873191/lma_M00741.args) Propanoyl-CoA metabolism, propanoyl-CoA => succinyl-CoA ([1](javascript:display('lma_M00741')))

[lma_M00307](https://www.genome.jp/kegg-bin/show_module?161298619873191/lma_M00307.args) Pyruvate oxidation, pyruvate => acetyl-CoA ([1](javascript:display('lma_M00307')))

[lma_M00034](https://www.genome.jp/kegg-bin/show_module?161298619873191/lma_M00034.args) Methionine salvage pathway ([1](javascript:display('lma_M00034')))

[lma_M00913](https://www.genome.jp/kegg-bin/show_module?161298619873191/lma_M00913.args) Pantothenate biosynthesis, 2-oxoisovalerate/spermine => pantothenate ([1](javascript:display('lma_M00913')))

[lma_M00135](https://www.genome.jp/kegg-bin/show_module?161298619873191/lma_M00135.args) GABA biosynthesis, eukaryotes, putrescine => GABA ([1](javascript:display('lma_M00135')))

**List of upregulated proteins at FDR = 0.05 and s0 = 0.4 in the N2 KO**

| [lma:LMJF_01_0780](https://www.kegg.jp/entry/lma:LMJF_01_0780) | putative eukaryotic initiation factor 4a |
| --- | --- |
| [lma:LMJF_24_2080](https://www.kegg.jp/entry/lma:LMJF_24_2080) | putative 40S ribosomal protein S8 |
| [lma:LMJF_23_0270](https://www.kegg.jp/entry/lma:LMJF_23_0270) | PTR1; pteridine reductase 1 |
| [lma:LMJF_35_0420](https://www.kegg.jp/entry/lma:LMJF_35_0420) | putative 40S ribosomal protein S3A |
| [lma:LMJF_36_5120](https://www.kegg.jp/entry/lma:LMJF_36_5120) | putative 40S ribosomal protein SA |
| [lma:LMJF_32_0370](https://www.kegg.jp/entry/lma:LMJF_32_0370) | hypothetical protein |
| [lma:LMJF_36_4510](https://www.kegg.jp/entry/lma:LMJF_36_4510) | RPL18; putative 60S ribosomal protein L18 |
| [lma:LMJF_10_0510](https://www.kegg.jp/entry/lma:LMJF_10_0510) | GPD; glycerol-3-phosphate dehydrogenase [NAD+],glycosomal |
| [lma:LMJF_35_2010](https://www.kegg.jp/entry/lma:LMJF_35_2010) | putative 40S ribosomal protein S6 |
| [lma:LMJF_01_0420](https://www.kegg.jp/entry/lma:LMJF_01_0420) | putative ribosomal protein S7 |
| [lma:LMJF_01_0520](https://www.kegg.jp/entry/lma:LMJF_01_0520) | putative long-chain-fatty-acid-CoA ligase |
| [lma:LMJF_01_0650](https://www.kegg.jp/entry/lma:LMJF_01_0650) | putative mitochondrial processing peptide beta subunit |
| [lma:LMJF_02_0290](https://www.kegg.jp/entry/lma:LMJF_02_0290) | putative protein kinase |
| [lma:LMJF_02_0450](https://www.kegg.jp/entry/lma:LMJF_02_0450) | putative voltage-dependent anion-selective channel |
| [lma:LMJF_02_0670](https://www.kegg.jp/entry/lma:LMJF_02_0670) | putative mitochondrial carrier protein |
| [lma:LMJF_03_0440](https://www.kegg.jp/entry/lma:LMJF_03_0440) | putative 60S acidic ribosomal protein P2 |
| [lma:LMJF_03_0520](https://www.kegg.jp/entry/lma:LMJF_03_0520) | hypothetical protein |
| [lma:LMJF_03_0690](https://www.kegg.jp/entry/lma:LMJF_03_0690) | hypothetical protein |
| [lma:LMJF_03_0980](https://www.kegg.jp/entry/lma:LMJF_03_0980) | putative elongation initiation factor 2 alpha subunit |
| [lma:LMJF_27_2330](https://www.kegg.jp/entry/lma:LMJF_27_2330) | putative GTP binding protein |
| [lma:LMJF_27_2360](https://www.kegg.jp/entry/lma:LMJF_27_2360) | hypothetical protein |
| [lma:LMJF_27_0700](https://www.kegg.jp/entry/lma:LMJF_27_0700) | hypothetical protein |
| [lma:LMJF_27_1250](https://www.kegg.jp/entry/lma:LMJF_27_1250) | hypothetical protein |
| [lma:LMJF_27_1310](https://www.kegg.jp/entry/lma:LMJF_27_1310) | putative arginyl-tRNA synthetase |
| [lma:LMJF_27_1390](https://www.kegg.jp/entry/lma:LMJF_27_1390) | putative 60S acidic ribosomal subunit protein |
| [lma:LMJF_27_1980](https://www.kegg.jp/entry/lma:LMJF_27_1980) | putative FtsJ cell division protein |
| [lma:LMJF_27_2020](https://www.kegg.jp/entry/lma:LMJF_27_2020) | D-lactate dehydrogenase-like protein |
| [lma:LMJF_27_2100](https://www.kegg.jp/entry/lma:LMJF_27_2100) | putative RNA-binding protein |
| [lma:LMJF_29_1090](https://www.kegg.jp/entry/lma:LMJF_29_1090) | putative ribosomal protein L1a |
| [lma:LMJF_29_1470](https://www.kegg.jp/entry/lma:LMJF_29_1470) | hypothetical protein |
| [lma:LMJF_29_2470](https://www.kegg.jp/entry/lma:LMJF_29_2470) | putative 60S ribosomal protein L13 |
| [lma:LMJF_29_2630](https://www.kegg.jp/entry/lma:LMJF_29_2630) | hypothetical protein |
| [lma:LMJF_29_2830](https://www.kegg.jp/entry/lma:LMJF_29_2830) | hypothetical protein |
| [lma:LMJF_29_2860](https://www.kegg.jp/entry/lma:LMJF_29_2860) | 40S ribosomal protein S19-like protein |
| [lma:LMJF_35_0240](https://www.kegg.jp/entry/lma:LMJF_35_0240) | 60S ribosomal protein L30 |
| [lma:LMJF_35_1900](https://www.kegg.jp/entry/lma:LMJF_35_1900) | putative 60S ribosomal protein L5 |
| [lma:LMJF_21_0730](https://www.kegg.jp/entry/lma:LMJF_21_0730) | putative 60S Ribosomal protein L36 |
| [lma:LMJF_35_1990](https://www.kegg.jp/entry/lma:LMJF_35_1990) | hypothetical protein |
| [lma:LMJF_35_2050](https://www.kegg.jp/entry/lma:LMJF_35_2050) | 60S ribosomal protein L32 |
| [lma:LMJF_35_2080](https://www.kegg.jp/entry/lma:LMJF_35_2080) | putative calcium motive p-type ATPase |
| [lma:LMJF_35_3080](https://www.kegg.jp/entry/lma:LMJF_35_3080) | putative glycerol kinase, glycosomal |
| [lma:LMJF_35_3100](https://www.kegg.jp/entry/lma:LMJF_35_3100) | putative ATP-dependent RNA helicase |
| [lma:LMJF_35_3290](https://www.kegg.jp/entry/lma:LMJF_35_3290) | putative 60S ribosomal subunit protein L31 |
| [lma:LMJF_35_3370](https://www.kegg.jp/entry/lma:LMJF_35_3370) | hypothetical protein |
| [lma:LMJF_35_3780](https://www.kegg.jp/entry/lma:LMJF_35_3780) | putative 60S ribosomal protein L27A/L29 |
| [lma:LMJF_35_3800](https://www.kegg.jp/entry/lma:LMJF_35_3800) | putative 60S ribosomal protein L23 |
| [lma:LMJF_35_3860](https://www.kegg.jp/entry/lma:LMJF_35_3860) | putative T-complex protein 1, eta subunit |
| [lma:LMJF_35_4130](https://www.kegg.jp/entry/lma:LMJF_35_4130) | PABP2; poly(A)-binding protein 2 |
| [lma:LMJF_35_4470](https://www.kegg.jp/entry/lma:LMJF_35_4470) | hypothetical protein |
| [lma:LMJF_35_4860](https://www.kegg.jp/entry/lma:LMJF_35_4860) | hypothetical protein |
| [lma:LMJF_35_4940](https://www.kegg.jp/entry/lma:LMJF_35_4940) | hypothetical protein |
| [lma:LMJF_35_5040](https://www.kegg.jp/entry/lma:LMJF_35_5040) | PABP1; polyadenylate-binding protein 1 |
| [lma:LMJF_04_0950](https://www.kegg.jp/entry/lma:LMJF_04_0950) | putative 60S ribosomal protein L10 |
| [lma:LMJF_35_1440](https://www.kegg.jp/entry/lma:LMJF_35_1440) | putative 60S ribosomal protein L2 |
| [lma:LMJF_36_6980](https://www.kegg.jp/entry/lma:LMJF_36_6980) | putative eukaryotic translation initiation factor 3 subunit 8 |
| [lma:LMJF_36_6160](https://www.kegg.jp/entry/lma:LMJF_36_6160) | hypothetical protein |
| [lma:LMJF_36_6060](https://www.kegg.jp/entry/lma:LMJF_36_6060) | putative eukaryotic initiation factor 4a |
| [lma:LMJF_36_5390](https://www.kegg.jp/entry/lma:LMJF_36_5390) | putative phosphoribosylpyrophosphate synthetase |
| [lma:LMJF_36_5100](https://www.kegg.jp/entry/lma:LMJF_36_5100) | hypothetical protein |
| [lma:LMJF_36_4420](https://www.kegg.jp/entry/lma:LMJF_36_4420) | putative 60S ribosomal protein L22 |
| [lma:LMJF_36_3880](https://www.kegg.jp/entry/lma:LMJF_36_3880) | putative eukaryotic translation initiation factor 3 subunit |
| [lma:LMJF_36_3840](https://www.kegg.jp/entry/lma:LMJF_36_3840) | putative glycyl tRNA synthetase |
| [lma:LMJF_36_3770](https://www.kegg.jp/entry/lma:LMJF_36_3770) | putative basic transcription factor 3a |
| [lma:LMJF_36_3750](https://www.kegg.jp/entry/lma:LMJF_36_3750) | putative 40S ribosomal protein S27-1 |
| [lma:LMJF_36_3070](https://www.kegg.jp/entry/lma:LMJF_36_3070) | fibrillarin |
| [lma:LMJF_36_2870](https://www.kegg.jp/entry/lma:LMJF_36_2870) | S24E-2; 40S ribosomal protein S24e |
| [lma:LMJF_36_2360](https://www.kegg.jp/entry/lma:LMJF_36_2360) | TAT; tyrosine aminotransferase |
| [lma:LMJF_07_0680](https://www.kegg.jp/entry/lma:LMJF_07_0680) | putative 40S ribosomal protein S9 |
| [lma:LMJF_36_1100](https://www.kegg.jp/entry/lma:LMJF_36_1100) | putative ribosomal protein L24 |
| [lma:LMJF_36_0990](https://www.kegg.jp/entry/lma:LMJF_36_0990) | putative 40S ribosomal protein S10 |
| [lma:LMJF_36_0940](https://www.kegg.jp/entry/lma:LMJF_36_0940) | putative 40S ribosomal protein S18 |
| [lma:LMJF_36_0250](https://www.kegg.jp/entry/lma:LMJF_36_0250) | EIF3-interacting protein-like protein |
| [lma:LMJF_36_0190](https://www.kegg.jp/entry/lma:LMJF_36_0190) | EF2-2; elongation factor 2 |
| [lma:LMJF_36_0050](https://www.kegg.jp/entry/lma:LMJF_36_0050) | PUF1; putative PUF1 |
| [lma:LMJF_34_3980](https://www.kegg.jp/entry/lma:LMJF_34_3980) | nucleolar protein family a member-like protein |
| [lma:LMJF_34_3650](https://www.kegg.jp/entry/lma:LMJF_34_3650) | putative 60S ribosomal protein L21 |
| [lma:LMJF_34_2900](https://www.kegg.jp/entry/lma:LMJF_34_2900) | putative ribosomal protein L3 |
| [lma:LMJF_34_2790](https://www.kegg.jp/entry/lma:LMJF_34_2790) | hypothetical protein |
| [lma:LMJF_34_1410](https://www.kegg.jp/entry/lma:LMJF_34_1410) | D-isomer specific 2-hydroxyacid dehydrogenase-like protein |
| [lma:LMJF_34_0840](https://www.kegg.jp/entry/lma:LMJF_34_0840) | putative translation elongation factor 1-beta |
| [lma:LMJF_25_1190](https://www.kegg.jp/entry/lma:LMJF_25_1190) | ribosomal protein S25 |
| [lma:LMJF_34_0110](https://www.kegg.jp/entry/lma:LMJF_34_0110) | putative adenylate kinase |
| [lma:LMJF_33_3150](https://www.kegg.jp/entry/lma:LMJF_33_3150) | putative 40S ribosomal protein S13 |
| [lma:LMJF_33_2810](https://www.kegg.jp/entry/lma:LMJF_33_2810) | transcription elongation factor-like protein |
| [lma:LMJF_33_2740](https://www.kegg.jp/entry/lma:LMJF_33_2740) | putative translation initiation factor IF-2 |
| [lma:LMJF_33_2390](https://www.kegg.jp/entry/lma:LMJF_33_2390) | putative heat shock protein |
| [lma:LMJF_33_0920](https://www.kegg.jp/entry/lma:LMJF_33_0920) | putative 40S ribosomal protein S3 |
| [lma:LMJF_33_0720](https://www.kegg.jp/entry/lma:LMJF_33_0720) | putative 60S ribosomal protein L6 |
| [lma:LMJF_33_0700](https://www.kegg.jp/entry/lma:LMJF_33_0700) | hypothetical protein |
| [lma:LMJF_33_0570](https://www.kegg.jp/entry/lma:LMJF_33_0570) | hypothetical protein |
| [lma:LMJF_32_2710](https://www.kegg.jp/entry/lma:LMJF_32_2710) | putative ribosomal protein L27 |
| [lma:LMJF_32_2150](https://www.kegg.jp/entry/lma:LMJF_32_2150) | hypothetical protein |
| [lma:LMJF_32_1000](https://www.kegg.jp/entry/lma:LMJF_32_1000) | putative chaperonin containing t-complex protein |
| [lma:LMJF_32_0950](https://www.kegg.jp/entry/lma:LMJF_32_0950) | hypothetical protein |
| [lma:LMJF_35_0600](https://www.kegg.jp/entry/lma:LMJF_35_0600) | putative 60S ribosomal protein L18a |
| [lma:LMJF_32_0750](https://www.kegg.jp/entry/lma:LMJF_32_0750) | putative RNA binding protein |
| [lma:LMJF_32_0450](https://www.kegg.jp/entry/lma:LMJF_32_0450) | 40S ribosomal protein S2 |
| [lma:LMJF_24_0040](https://www.kegg.jp/entry/lma:LMJF_24_0040) | putative 60S ribosomal protein L17 |
| [lma:LMJF_32_0400](https://www.kegg.jp/entry/lma:LMJF_32_0400) | putative ATP-dependent RNA helicase |
| [lma:LMJF_32_0020](https://www.kegg.jp/entry/lma:LMJF_32_0020) | hypothetical protein |
| [lma:LMJF_31_2970](https://www.kegg.jp/entry/lma:LMJF_31_2970) | putative acetyl-CoA carboxylase |
| [lma:LMJF_31_1750](https://www.kegg.jp/entry/lma:LMJF_31_1750) | nucleosome assembly protein-like protein |
| [lma:LMJF_31_1130](https://www.kegg.jp/entry/lma:LMJF_31_1130) | putative N-acyl-L-amino acid amidohydrolase |
| [lma:LMJF_31_0160](https://www.kegg.jp/entry/lma:LMJF_31_0160) | putative DNA-directed RNA polymerase II subunit 2 |
| [lma:LMJF_31_0050](https://www.kegg.jp/entry/lma:LMJF_31_0050) | CYP5; putative cyclophilin 5 |
| [lma:LMJF_30_3720](https://www.kegg.jp/entry/lma:LMJF_30_3720) | LIP1; putative 60S acidic ribosomal protein P2 |
| [lma:LMJF_28_0960](https://www.kegg.jp/entry/lma:LMJF_28_0960) | 40S ribosomal protein S14 |
| [lma:LMJF_30_3520](https://www.kegg.jp/entry/lma:LMJF_30_3520) | METK2; S-adenosylmethionine synthetase |
| [lma:LMJF_30_3450](https://www.kegg.jp/entry/lma:LMJF_30_3450) | hypothetical protein |
| [lma:LMJF_30_3430](https://www.kegg.jp/entry/lma:LMJF_30_3430) | hypothetical protein |
| [lma:LMJF_21_1050](https://www.kegg.jp/entry/lma:LMJF_21_1050) | putative 60S ribosomal protein L9 |
| [lma:LMJF_30_3240](https://www.kegg.jp/entry/lma:LMJF_30_3240) | putative glutamyl-tRNA synthetase |
| [lma:LMJF_30_3090](https://www.kegg.jp/entry/lma:LMJF_30_3090) | hypothetical protein |
| [lma:LMJF_30_3040](https://www.kegg.jp/entry/lma:LMJF_30_3040) | eukaryotic translation initiation factor 3 subunit 7-like protein |
| [lma:LMJF_30_2630](https://www.kegg.jp/entry/lma:LMJF_30_2630) | putative replication factor C, subunit 2 |
| [lma:LMJF_30_1120](https://www.kegg.jp/entry/lma:LMJF_30_1120) | putative importin alpha |
| [lma:LMJF_30_0730](https://www.kegg.jp/entry/lma:LMJF_30_0730) | putative co-chaperone GrpE |
| [lma:LMJF_30_0460](https://www.kegg.jp/entry/lma:LMJF_30_0460) | putative aspartyl-tRNA synthetase |
| [lma:LMJF_30_0180](https://www.kegg.jp/entry/lma:LMJF_30_0180) | putative 2-hydroxy-3-oxopropionate reductase |
| [lma:LMJF_28_2750](https://www.kegg.jp/entry/lma:LMJF_28_2750) | LACK2; activated protein kinase C receptor (LACK) |
| [lma:LMJF_28_2170](https://www.kegg.jp/entry/lma:LMJF_28_2170) | hypothetical protein |
| [lma:LMJF_28_0540](https://www.kegg.jp/entry/lma:LMJF_28_0540) | putative ribosomal protein S26 |
| [lma:LMJF_26_0890](https://www.kegg.jp/entry/lma:LMJF_26_0890) | putative 40S ribosomal protein S16 |
| [lma:LMJF_26_0840](https://www.kegg.jp/entry/lma:LMJF_26_0840) | hypothetical protein |
| [lma:LMJF_26_0620](https://www.kegg.jp/entry/lma:LMJF_26_0620) | putative 10 kDa heat shock protein |
| [lma:LMJF_26_0380](https://www.kegg.jp/entry/lma:LMJF_26_0380) | hypothetical protein |
| [lma:LMJF_26_0180](https://www.kegg.jp/entry/lma:LMJF_26_0180) | putative 60S ribosomal protein L7 |
| [lma:LMJF_25_1840](https://www.kegg.jp/entry/lma:LMJF_25_1840) | hypothetical protein |
| [lma:LMJF_25_1610](https://www.kegg.jp/entry/lma:LMJF_25_1610) | hypothetical protein |
| [lma:LMJF_25_1080](https://www.kegg.jp/entry/lma:LMJF_25_1080) | hypothetical protein |
| [lma:LMJF_25_0730](https://www.kegg.jp/entry/lma:LMJF_25_0730) | EIF5A2; putative eukaryotic initiation factor 5a |
| [lma:LMJF_25_0080](https://www.kegg.jp/entry/lma:LMJF_25_0080) | PABP3; poly(A)-binding protein 3 |
| [lma:LMJF_35_1670](https://www.kegg.jp/entry/lma:LMJF_35_1670) | putative 60S ribosomal protein L26 |
| [lma:LMJF_24_0770](https://www.kegg.jp/entry/lma:LMJF_24_0770) | putative malic enzyme |
| [lma:LMJF_24_0520](https://www.kegg.jp/entry/lma:LMJF_24_0520) | putative DNAJ domain protein |
| [lma:LMJF_24_0370](https://www.kegg.jp/entry/lma:LMJF_24_0370) | putative aspartate aminotransferase |
| [lma:LMJF_23_0080](https://www.kegg.jp/entry/lma:LMJF_23_0080) | hypothetical protein |
| [lma:LMJF_23_0050](https://www.kegg.jp/entry/lma:LMJF_23_0050) | CYP11; putative cyclophilin 11 |
| [lma:LMJF_22_1600](https://www.kegg.jp/entry/lma:LMJF_22_1600) | putative ser/thr protein phosphatase |
| [lma:LMJF_22_1560](https://www.kegg.jp/entry/lma:LMJF_22_1560) | putative 40S ribosomal protein L14 |
| [lma:LMJF_22_0470](https://www.kegg.jp/entry/lma:LMJF_22_0470) | hypothetical protein |
| [lma:LMJF_22_0460](https://www.kegg.jp/entry/lma:LMJF_22_0460) | putative 40S ribosomal protein S15 |
| [lma:LMJF_22_0300](https://www.kegg.jp/entry/lma:LMJF_22_0300) | hypothetical protein |
| [lma:LMJF_22_0110](https://www.kegg.jp/entry/lma:LMJF_22_0110) | putative GMP synthase |
| [lma:LMJF_22_0080](https://www.kegg.jp/entry/lma:LMJF_22_0080) | putative heat shock protein DNAJ |
| [lma:LMJF_21_1300](https://www.kegg.jp/entry/lma:LMJF_21_1300) | hypothetical protein |
| [lma:LMJF_21_1250](https://www.kegg.jp/entry/lma:LMJF_21_1250) | putative adenylate kinase |
| [lma:LMJF_21_1070](https://www.kegg.jp/entry/lma:LMJF_21_1070) | putative 40S ribosomal protein S23 |
| [lma:LMJF_21_0810](https://www.kegg.jp/entry/lma:LMJF_21_0810) | putative methionyl-tRNA synthetase |
| [lma:LMJF_21_0800](https://www.kegg.jp/entry/lma:LMJF_21_0800) | hypothetical protein |
| [lma:LMJF_21_0540](https://www.kegg.jp/entry/lma:LMJF_21_0540) | putative la RNA binding protein |
| [lma:LMJF_21_0430](https://www.kegg.jp/entry/lma:LMJF_21_0430) | hypothetical protein |
| [lma:LMJF_21_1550](https://www.kegg.jp/entry/lma:LMJF_21_1550) | putative 40S ribosomal protein S11 |
| [lma:LMJF_20_1290](https://www.kegg.jp/entry/lma:LMJF_20_1290) | hypothetical protein |
| [lma:LMJF_20_1130](https://www.kegg.jp/entry/lma:LMJF_20_1130) | DNAj-like protein |
| [lma:LMJF_20_0650](https://www.kegg.jp/entry/lma:LMJF_20_0650) | rRNA biogenesis protein-like protein |
| [lma:LMJF_19_0440](https://www.kegg.jp/entry/lma:LMJF_19_0440) | putative nucleosome assembly protein |
| [lma:LMJF_19_0160](https://www.kegg.jp/entry/lma:LMJF_19_0160) | putative aminopeptidase |
| [lma:LMJF_19_0100](https://www.kegg.jp/entry/lma:LMJF_19_0100) | putative fibrillarin |
| [lma:LMJF_19_0090](https://www.kegg.jp/entry/lma:LMJF_19_0090) | hypothetical protein |
| [lma:LMJF_18_1510](https://www.kegg.jp/entry/lma:LMJF_18_1510) | H1A-1; putative P-type H+-ATPase |
| [lma:LMJF_18_0940](https://www.kegg.jp/entry/lma:LMJF_18_0940) | hypothetical protein |
| [lma:LMJF_18_0820](https://www.kegg.jp/entry/lma:LMJF_18_0820) | hypothetical protein |
| [lma:LMJF_36_3760](https://www.kegg.jp/entry/lma:LMJF_36_3760) | RPL10a; putative 60S ribosomal protein L10a |
| [lma:LMJF_18_0230](https://www.kegg.jp/entry/lma:LMJF_18_0230) | putative 60S ribosomal protein L7 |
| [lma:LMJF_17_1290](https://www.kegg.jp/entry/lma:LMJF_17_1290) | putative translation initiation factor |
| [lma:LMJF_17_0980](https://www.kegg.jp/entry/lma:LMJF_17_0980) | hypothetical protein |
| [lma:LMJF_17_0550](https://www.kegg.jp/entry/lma:LMJF_17_0550) | putative RNA-binding protein |
| [lma:LMJF_17_0250](https://www.kegg.jp/entry/lma:LMJF_17_0250) | CYSB; cystathionine beta-synthase |
| [lma:LMJF_17_0010](https://www.kegg.jp/entry/lma:LMJF_17_0010) | hypothetical protein |
| [lma:LMJF_16_1600](https://www.kegg.jp/entry/lma:LMJF_16_1600) | EIF4G3; putative eukaryotic translation initiation factor 4 gamma |
| [lma:LMJF_16_0550](https://www.kegg.jp/entry/lma:LMJF_16_0550) | putative OMPDCase-OPRTase |
| [lma:LMJF_15_1470](https://www.kegg.jp/entry/lma:LMJF_15_1470) | putative ribosomal protein S6 |
| [lma:LMJF_15_1380](https://www.kegg.jp/entry/lma:LMJF_15_1380) | putative nucleolar RNA binding protein |
| [lma:LMJF_15_1207](https://www.kegg.jp/entry/lma:LMJF_15_1207) | 60S acidic ribosomal protein P2 |
| [lma:LMJF_15_0380](https://www.kegg.jp/entry/lma:LMJF_15_0380) | putative 60S acidic ribosomal protein |
| [lma:LMJF_15_0230](https://www.kegg.jp/entry/lma:LMJF_15_0230) | putative lysyl-tRNA synthetase |
| [lma:LMJF_14_0190](https://www.kegg.jp/entry/lma:LMJF_14_0190) | hypothetical protein |
| [lma:LMJF_13_1230](https://www.kegg.jp/entry/lma:LMJF_13_1230) | putative 40S ribosomal protein S4 |
| [lma:LMJF_13_0680](https://www.kegg.jp/entry/lma:LMJF_13_0680) | hypothetical protein |
| [lma:LMJF_13_0570](https://www.kegg.jp/entry/lma:LMJF_13_0570) | putative 40S ribosomal protein S12 |
| [lma:LMJF_29_1800](https://www.kegg.jp/entry/lma:LMJF_29_1800) | putative 40S ribosomal protein S15A |
| [lma:LMJF_11_1160](https://www.kegg.jp/entry/lma:LMJF_11_1160) | putative protein transport protein Sec31 |
| [lma:LMJF_11_1130](https://www.kegg.jp/entry/lma:LMJF_11_1130) | putative 60S ribosomal protein L28 |
| [lma:LMJF_11_1100](https://www.kegg.jp/entry/lma:LMJF_11_1100) | putative lanosterol 14-alpha-demethylase |
| [lma:LMJF_10_0465](https://www.kegg.jp/entry/lma:LMJF_10_0465) | GP63-2; GP63, leishmanolysin |
| [lma:LMJF_10_0210](https://www.kegg.jp/entry/lma:LMJF_10_0210) | putative nucleolar protein |
| [lma:LMJF_34_2470](https://www.kegg.jp/entry/lma:LMJF_34_2470) | putative ribosomal protein l35a |
| [lma:LMJF_09_1220](https://www.kegg.jp/entry/lma:LMJF_09_1220) | putative AAA family ATPase |
| [lma:LMJF_09_1070](https://www.kegg.jp/entry/lma:LMJF_09_1070) | putative eukaryotic translation initiation factor 2 subunit |
| [lma:LMJF_08_0550](https://www.kegg.jp/entry/lma:LMJF_08_0550) | translation initiation factor-like protein |
| [lma:LMJF_07_0850](https://www.kegg.jp/entry/lma:LMJF_07_0850) | putative ubiquitin carrier protein |
| [lma:LMJF_07_0640](https://www.kegg.jp/entry/lma:LMJF_07_0640) | hypothetical protein |
| [lma:LMJF_07_0510](https://www.kegg.jp/entry/lma:LMJF_07_0510) | putative 60S ribosomal protein L7a |
| [lma:LMJF_07_0340](https://www.kegg.jp/entry/lma:LMJF_07_0340) | putative ATP-dependent DEAD/H RNA helicase |
| [lma:LMJF_06_0580](https://www.kegg.jp/entry/lma:LMJF_06_0580) | putative 60S ribosomal protein L23a |
| [lma:LMJF_06_0415](https://www.kegg.jp/entry/lma:LMJF_06_0415) | putative 60S ribosomal protein L19 |
| [lma:LMJF_05_0140](https://www.kegg.jp/entry/lma:LMJF_05_0140) | putative nucleolar RNA helicase II |
| [lma:LMJF_20_1280](https://www.kegg.jp/entry/lma:LMJF_20_1280) | SMP-4; putative small myristoylated protein 4 |
| [lma:LMJF_09_0970](https://www.kegg.jp/entry/lma:LMJF_09_0970) | EF1G; elongation factor-1 gamma |
| [lma:LMJF_36_1430](https://www.kegg.jp/entry/lma:LMJF_36_1430) | putative translation elongation factor 1-beta |
| [lma:LMJF_28_1280](https://www.kegg.jp/entry/lma:LMJF_28_1280) | PAH; phenylalanine-4-hydroxylase |
| [lma:LMJF_11_0970](https://www.kegg.jp/entry/lma:LMJF_11_0970) | 40S ribosomal protein S5 |
| [lma:LMJF_31_1010](https://www.kegg.jp/entry/lma:LMJF_31_1010) | hypothetical protein |
| [lma:LMJF_04_0990](https://www.kegg.jp/entry/lma:LMJF_04_0990) | hypothetical protein |
| [lma:LMJF_04_0770](https://www.kegg.jp/entry/lma:LMJF_04_0770) | nascent polypeptide associated complex subunit-like protein, copy 1 |

**List of downregulated proteins at FDR = 0.05 and s0 = 0.4 in the N2 KO**

| [lma:LMJF_29_1960](https://www.kegg.jp/entry/lma:LMJF_29_1960) | putative fumarate hydratase |
| --- | --- |
| [lma:LMJF_31_2150](https://www.kegg.jp/entry/lma:LMJF_31_2150) | PGFS; prostaglandin F2-alpha synthase/D-arabinose dehydrogenase |
| [lma:LMJF_20_0970](https://www.kegg.jp/entry/lma:LMJF_20_0970) | putative 1,2-Dihydroxy-3-keto-5-methylthiopentene dioxygenase |
| [lma:LMJF_18_0680](https://www.kegg.jp/entry/lma:LMJF_18_0680) | putative citrate synthase |
| [lma:LMJF_17_0725](https://www.kegg.jp/entry/lma:LMJF_17_0725) | guanosine monophosphate reductase |
| [lma:LMJF_27_2660](https://www.kegg.jp/entry/lma:LMJF_27_2660) | putative peptidyl dipeptidase |
| [lma:LMJF_03_0230](https://www.kegg.jp/entry/lma:LMJF_03_0230) | putative long chain fatty Acyl CoA synthetase |
| [lma:LMJF_03_0570](https://www.kegg.jp/entry/lma:LMJF_03_0570) | putative quinone oxidoreductase |
| [lma:LMJF_27_0500](https://www.kegg.jp/entry/lma:LMJF_27_0500) | putative calpain-like cysteine peptidase |
| [lma:LMJF_27_0760](https://www.kegg.jp/entry/lma:LMJF_27_0760) | putative small GTP-binding protein Rab1 |
| [lma:LMJF_27_0930](https://www.kegg.jp/entry/lma:LMJF_27_0930) | putative isovaleryl-coA dehydrogenase |
| [lma:LMJF_27_1060](https://www.kegg.jp/entry/lma:LMJF_27_1060) | putative cysteine desulfurase |
| [lma:LMJF_27_1805](https://www.kegg.jp/entry/lma:LMJF_27_1805) | putative glycosomal phosphoenolpyruvate carboxykinase |
| [lma:LMJF_27_2030](https://www.kegg.jp/entry/lma:LMJF_27_2030) | putative branched-chain amino acid aminotransferase |
| [lma:LMJF_29_1160](https://www.kegg.jp/entry/lma:LMJF_29_1160) | TXN1; tryparedoxin |
| [lma:LMJF_29_1270](https://www.kegg.jp/entry/lma:LMJF_29_1270) | putative serine peptidase |
| [lma:LMJF_29_1310](https://www.kegg.jp/entry/lma:LMJF_29_1310) | putative carnitine/choline acetyltransferase |
| [lma:LMJF_29_1720](https://www.kegg.jp/entry/lma:LMJF_29_1720) | putative histone H2A |
| [lma:LMJF_29_1770](https://www.kegg.jp/entry/lma:LMJF_29_1770) | putative paraflagellar rod protein 1D |
| [lma:LMJF_29_2310](https://www.kegg.jp/entry/lma:LMJF_29_2310) | enoyl-CoA hydratase/isomerase-like protein |
| [lma:LMJF_29_2450](https://www.kegg.jp/entry/lma:LMJF_29_2450) | putative heat shock protein 20 |
| [lma:LMJF_29_2570](https://www.kegg.jp/entry/lma:LMJF_29_2570) | putative serine/threonine-protein kinase |
| [lma:LMJF_35_0030](https://www.kegg.jp/entry/lma:LMJF_35_0030) | pyruvate kinase |
| [lma:LMJF_35_0970](https://www.kegg.jp/entry/lma:LMJF_35_0970) | putative aldose 1-epimerase |
| [lma:LMJF_35_1180](https://www.kegg.jp/entry/lma:LMJF_35_1180) | putative NADH-dependent fumarate reductase |
| [lma:LMJF_35_1230](https://www.kegg.jp/entry/lma:LMJF_35_1230) | putative short chain dehydrogenase |
| [lma:LMJF_35_2420](https://www.kegg.jp/entry/lma:LMJF_35_2420) | putative phosphoinositide-binding protein |
| [lma:LMJF_35_2440](https://www.kegg.jp/entry/lma:LMJF_35_2440) | hypothetical protein |
| [lma:LMJF_35_3070](https://www.kegg.jp/entry/lma:LMJF_35_3070) | hypothetical protein |
| [lma:LMJF_35_3840](https://www.kegg.jp/entry/lma:LMJF_35_3840) | putative proteasome beta 2 subunit |
| [lma:LMJF_36_6650](https://www.kegg.jp/entry/lma:LMJF_36_6650) | PGAM; 2,3-bisphosphoglycerate-independent phosphoglycerate mutase |
| [lma:LMJF_36_6540](https://www.kegg.jp/entry/lma:LMJF_36_6540) | similarity to endo-1-like protein |
| [lma:LMJF_36_6180](https://www.kegg.jp/entry/lma:LMJF_36_6180) | hypothetical protein |
| [lma:LMJF_36_5360](https://www.kegg.jp/entry/lma:LMJF_36_5360) | putative ubiquinone biosynthesis methyltransferase |
| [lma:LMJF_36_5130](https://www.kegg.jp/entry/lma:LMJF_36_5130) | hypothetical protein |
| [lma:LMJF_36_3590](https://www.kegg.jp/entry/lma:LMJF_36_3590) | cysteine synthase |
| [lma:LMJF_36_1960](https://www.kegg.jp/entry/lma:LMJF_36_1960) | PMM; putative phosphomannomutase |
| [lma:LMJF_36_1630](https://www.kegg.jp/entry/lma:LMJF_36_1630) | putative clathrin heavy chain |
| [lma:LMJF_36_1260](https://www.kegg.jp/entry/lma:LMJF_36_1260) | ALD; fructose-1,6-bisphosphate aldolase |
| [lma:LMJF_36_1140](https://www.kegg.jp/entry/lma:LMJF_36_1140) | putative short chain 3-hydroxyacyl-CoA dehydrogenase |
| [lma:LMJF_36_0740](https://www.kegg.jp/entry/lma:LMJF_36_0740) | hypothetical protein |
| [lma:LMJF_36_0230](https://www.kegg.jp/entry/lma:LMJF_36_0230) | putative peptidyl-prolyl cis-trans isomerase |
| [lma:LMJF_34_4310](https://www.kegg.jp/entry/lma:LMJF_34_4310) | putative coatomer alpha subunit |
| [lma:LMJF_34_3380](https://www.kegg.jp/entry/lma:LMJF_34_3380) | aldose 1-epimerase-like protein |
| [lma:LMJF_34_2610](https://www.kegg.jp/entry/lma:LMJF_34_2610) | putative ATP-dependent DNA helicase |
| [lma:LMJF_34_2540](https://www.kegg.jp/entry/lma:LMJF_34_2540) | hypothetical protein |
| [lma:LMJF_34_2510](https://www.kegg.jp/entry/lma:LMJF_34_2510) | protein phosphatase 2C-like protein |
| [lma:LMJF_34_0690](https://www.kegg.jp/entry/lma:LMJF_34_0690) | hypothetical protein |
| [lma:LMJF_34_0150](https://www.kegg.jp/entry/lma:LMJF_34_0150) | putative malate dehydrogenase |
| [lma:LMJF_34_0140](https://www.kegg.jp/entry/lma:LMJF_34_0140) | malate dehydrogenase |
| [lma:LMJF_34_0070](https://www.kegg.jp/entry/lma:LMJF_34_0070) | APX; ascorbate peroxidase |
| [lma:LMJF_33_2550](https://www.kegg.jp/entry/lma:LMJF_33_2550) | putative isocitrate dehydrogenase |
| [lma:LMJF_33_2340](https://www.kegg.jp/entry/lma:LMJF_33_2340) | putative succinyl-coA:3-ketoacid-coenzyme A transferase, mitochondrial precursor |
| [lma:LMJF_33_0830](https://www.kegg.jp/entry/lma:LMJF_33_0830) | putative 2,4-dienoyl-coa reductase fadh1 |
| [lma:LMJF_32_1830](https://www.kegg.jp/entry/lma:LMJF_32_1830) | SODB2; putative iron superoxide dismutase |
| [lma:LMJF_32_1730](https://www.kegg.jp/entry/lma:LMJF_32_1730) | putative coatomer epsilon subunit |
| [lma:LMJF_32_1260](https://www.kegg.jp/entry/lma:LMJF_32_1260) | hypothetical protein |
| [lma:LMJF_32_0630](https://www.kegg.jp/entry/lma:LMJF_32_0630) | hypothetical protein |
| [lma:LMJF_32_0360](https://www.kegg.jp/entry/lma:LMJF_32_0360) | hypothetical protein |
| [lma:LMJF_31_3160](https://www.kegg.jp/entry/lma:LMJF_31_3160) | putative protein kinase |
| [lma:LMJF_31_3130](https://www.kegg.jp/entry/lma:LMJF_31_3130) | methylcrotonoyl-coa carboxylase biotinylated subunitprotein-like protein |
| [lma:LMJF_31_3110](https://www.kegg.jp/entry/lma:LMJF_31_3110) | hypothetical protein |
| [lma:LMJF_31_2960](https://www.kegg.jp/entry/lma:LMJF_31_2960) | putative serine/threonine-protein kinase |
| [lma:LMJF_31_2880](https://www.kegg.jp/entry/lma:LMJF_31_2880) | putative aldehyde reductase |
| [lma:LMJF_31_2330](https://www.kegg.jp/entry/lma:LMJF_31_2330) | putative 3,2-trans-enoyl-CoA isomerase mitochondrial precursor |
| [lma:LMJF_31_2170](https://www.kegg.jp/entry/lma:LMJF_31_2170) | hypothetical protein |
| [lma:LMJF_31_1640](https://www.kegg.jp/entry/lma:LMJF_31_1640) | thiolase protein-like protein |
| [lma:LMJF_31_1220](https://www.kegg.jp/entry/lma:LMJF_31_1220) | putative vacuolar-type proton translocating pyrophosphatase 1 |
| [lma:LMJF_31_1210](https://www.kegg.jp/entry/lma:LMJF_31_1210) | hypothetical protein |
| [lma:LMJF_31_1140](https://www.kegg.jp/entry/lma:LMJF_31_1140) | putative monoglyceride lipase |
| [lma:LMJF_31_0390](https://www.kegg.jp/entry/lma:LMJF_31_0390) | putative calpain-like cysteine peptidase |
| [lma:LMJF_30_3680](https://www.kegg.jp/entry/lma:LMJF_30_3680) | hypothetical protein |
| [lma:LMJF_30_2970](https://www.kegg.jp/entry/lma:LMJF_30_2970) | glyceraldehyde 3-phosphate dehydrogenase,glycosomal |
| [lma:LMJF_30_2850](https://www.kegg.jp/entry/lma:LMJF_30_2850) | hypothetical protein |
| [lma:LMJF_30_2600](https://www.kegg.jp/entry/lma:LMJF_30_2600) | FTHS; formate--tetrahydrofolate ligase |
| [lma:LMJF_30_2330](https://www.kegg.jp/entry/lma:LMJF_30_2330) | hypothetical protein |
| [lma:LMJF_30_1940](https://www.kegg.jp/entry/lma:LMJF_30_1940) | succinyl-coa:3-ketoacid-coenzyme a transferase-like protein |
| [lma:LMJF_30_0930](https://www.kegg.jp/entry/lma:LMJF_30_0930) | hypothetical protein |
| [lma:LMJF_28_2910](https://www.kegg.jp/entry/lma:LMJF_28_2910) | putative glutamate dehydrogenase |
| [lma:LMJF_28_2860](https://www.kegg.jp/entry/lma:LMJF_28_2860) | cMDH; cytosolic malate dehydrogenase |
| [lma:LMJF_28_2510](https://www.kegg.jp/entry/lma:LMJF_28_2510) | putative acyl-CoA dehydrogenase |
| [lma:LMJF_28_2250](https://www.kegg.jp/entry/lma:LMJF_28_2250) | glycosomal membrane protein-like protein |
| [lma:LMJF_28_1570](https://www.kegg.jp/entry/lma:LMJF_28_1570) | putative hydrolase, alpha/beta fold family |
| [lma:LMJF_28_1380](https://www.kegg.jp/entry/lma:LMJF_28_1380) | haloacid dehalogenase-like hydrolase-like protein |
| [lma:LMJF_28_1230](https://www.kegg.jp/entry/lma:LMJF_28_1230) | hypothetical protein |
| [lma:LMJF_28_1140](https://www.kegg.jp/entry/lma:LMJF_28_1140) | putative electron-transfer-flavoprotein,alpha polypeptide |
| [lma:LMJF_28_0380](https://www.kegg.jp/entry/lma:LMJF_28_0380) | hypothetical protein |
| [lma:LMJF_26_2640](https://www.kegg.jp/entry/lma:LMJF_26_2640) | hypothetical protein |
| [lma:LMJF_26_2280](https://www.kegg.jp/entry/lma:LMJF_26_2280) | putative nitrilase |
| [lma:LMJF_26_1710](https://www.kegg.jp/entry/lma:LMJF_26_1710) | putative cytochrome c oxidase subunit V |
| [lma:LMJF_26_1550](https://www.kegg.jp/entry/lma:LMJF_26_1550) | trifunctional enzyme alpha subunit, mitochondrial precursor-like protein |
| [lma:LMJF_26_0810](https://www.kegg.jp/entry/lma:LMJF_26_0810) | TDPX; type II (glutathione peroxidase-like) tryparedoxin peroxidase |
| [lma:LMJF_26_0030](https://www.kegg.jp/entry/lma:LMJF_26_0030) | GCVP; putative glycine dehydrogenase |
| [lma:LMJF_26_0020](https://www.kegg.jp/entry/lma:LMJF_26_0020) | methylmalonyl-coa epimerase-like protein |
| [lma:LMJF_25_2410](https://www.kegg.jp/entry/lma:LMJF_25_2410) | hypothetical protein |
| [lma:LMJF_25_1820](https://www.kegg.jp/entry/lma:LMJF_25_1820) | hypothetical protein |
| [lma:LMJF_25_1120](https://www.kegg.jp/entry/lma:LMJF_25_1120) | ALDH2; aldehyde dehydrogenase, mitochondrial precursor |
| [lma:LMJF_25_1060](https://www.kegg.jp/entry/lma:LMJF_25_1060) | hypothetical protein |
| [lma:LMJF_25_0120](https://www.kegg.jp/entry/lma:LMJF_25_0120) | putative electron transfer flavoprotein |
| [lma:LMJF_24_2060](https://www.kegg.jp/entry/lma:LMJF_24_2060) | transketolase |
| [lma:LMJF_24_1740](https://www.kegg.jp/entry/lma:LMJF_24_1740) | hypothetical protein |
| [lma:LMJF_24_0850](https://www.kegg.jp/entry/lma:LMJF_24_0850) | triosephosphate isomerase |
| [lma:LMJF_23_1480](https://www.kegg.jp/entry/lma:LMJF_23_1480) | putative alanine racemase |
| [lma:LMJF_23_1000](https://www.kegg.jp/entry/lma:LMJF_23_1000) | hypothetical protein |
| [lma:LMJF_23_0260](https://www.kegg.jp/entry/lma:LMJF_23_0260) | putative argininosuccinate synthase |
| [lma:LMJF_23_0110](https://www.kegg.jp/entry/lma:LMJF_23_0110) | GDPMP; mannose-1-phosphate guanyltransferase |
| [lma:LMJF_23_0040](https://www.kegg.jp/entry/lma:LMJF_23_0040) | peroxidoxin |
| [lma:LMJF_22_0780](https://www.kegg.jp/entry/lma:LMJF_22_0780) | putative NADH-cytochrome b5 reductase |
| [lma:LMJF_22_0180](https://www.kegg.jp/entry/lma:LMJF_22_0180) | hypothetical protein |
| [lma:LMJF_21_1558](https://www.kegg.jp/entry/lma:LMJF_21_1558) | hypothetical protein |
| [lma:LMJF_21_0550](https://www.kegg.jp/entry/lma:LMJF_21_0550) | dihydrolipoamide acetyltransferase precursorlike protein |
| [lma:LMJF_20_0810](https://www.kegg.jp/entry/lma:LMJF_20_0810) | putative N-ethylmaleimide-sensitive factor |
| [lma:LMJF_20_0580](https://www.kegg.jp/entry/lma:LMJF_20_0580) | hypothetical protein |
| [lma:LMJF_20_0110](https://www.kegg.jp/entry/lma:LMJF_20_0110) | PGKB; phosphoglycerate kinase B, cytosolic |
| [lma:LMJF_19_1160](https://www.kegg.jp/entry/lma:LMJF_19_1160) | hypothetical protein |
| [lma:LMJF_19_1005](https://www.kegg.jp/entry/lma:LMJF_19_1005) | 4-coumarate:coa ligase-like protein |
| [lma:LMJF_19_0985](https://www.kegg.jp/entry/lma:LMJF_19_0985) | 4-coumarate:coa ligase-like protein |
| [lma:LMJF_19_0710](https://www.kegg.jp/entry/lma:LMJF_19_0710) | gMDH; glycosomal malate dehydrogenase |
| [lma:LMJF_19_0260](https://www.kegg.jp/entry/lma:LMJF_19_0260) | putative C-terminal motor kinesin |
| [lma:LMJF_19_0050](https://www.kegg.jp/entry/lma:LMJF_19_0050) | histone H2B |
| [lma:LMJF_18_1300](https://www.kegg.jp/entry/lma:LMJF_18_1300) | hypothetical protein |
| [lma:LMJF_18_0580](https://www.kegg.jp/entry/lma:LMJF_18_0580) | putative peroxisomal enoyl-coa hydratase |
| [lma:LMJF_17_0480](https://www.kegg.jp/entry/lma:LMJF_17_0480) | hypothetical protein |
| [lma:LMJF_16_1610](https://www.kegg.jp/entry/lma:LMJF_16_1610) | prohibitin |
| [lma:LMJF_16_1430](https://www.kegg.jp/entry/lma:LMJF_16_1430) | paraflagellar rod protein 2C |
| [lma:LMJF_16_0950](https://www.kegg.jp/entry/lma:LMJF_16_0950) | sucrose-phosphate synthase-like protein |
| [lma:LMJF_15_1480](https://www.kegg.jp/entry/lma:LMJF_15_1480) | putative cAMP specific phosphodiesterase |
| [lma:LMJF_15_1140](https://www.kegg.jp/entry/lma:LMJF_15_1140) | TRYP6; tryparedoxin peroxidase |
| [lma:LMJF_14_1480](https://www.kegg.jp/entry/lma:LMJF_14_1480) | putative glutathione-S-transferase/glutaredoxin |
| [lma:LMJF_14_1320](https://www.kegg.jp/entry/lma:LMJF_14_1320) | SHMT-S; serine hydroxymethyltranferase (SHMT-S) |
| [lma:LMJF_14_1160](https://www.kegg.jp/entry/lma:LMJF_14_1160) | ENOL; enolase |
| [lma:LMJF_14_1100](https://www.kegg.jp/entry/lma:LMJF_14_1100) | putative kinesin K39 |
| [lma:LMJF_14_0850](https://www.kegg.jp/entry/lma:LMJF_14_0850) | SMP-3; putative small myristoylated protein-3 |
| [lma:LMJF_14_0240](https://www.kegg.jp/entry/lma:LMJF_14_0240) | hypothetical protein |
| [lma:LMJF_14_0180](https://www.kegg.jp/entry/lma:LMJF_14_0180) | putative carboxypeptidase |
| [lma:LMJF_13_1680](https://www.kegg.jp/entry/lma:LMJF_13_1680) | P5CR; pyrroline-5-carboxylate reductase |
| [lma:LMJF_13_1650](https://www.kegg.jp/entry/lma:LMJF_13_1650) | putative dynein heavy chain |
| [lma:LMJF_13_1350](https://www.kegg.jp/entry/lma:LMJF_13_1350) | hypothetical protein |
| [lma:LMJF_13_1140](https://www.kegg.jp/entry/lma:LMJF_13_1140) | hypothetical protein |
| [lma:LMJF_13_1050](https://www.kegg.jp/entry/lma:LMJF_13_1050) | hypothetical protein |
| [lma:LMJF_13_0650](https://www.kegg.jp/entry/lma:LMJF_13_0650) | hypothetical protein |
| [lma:LMJF_13_0160](https://www.kegg.jp/entry/lma:LMJF_13_0160) | putative protein kinase A regulatory subunit |
| [lma:LMJF_13_0090](https://www.kegg.jp/entry/lma:LMJF_13_0090) | metallo-peptidase, Clan MA(E), family 32 |
| [lma:LMJF_12_1130](https://www.kegg.jp/entry/lma:LMJF_12_1130) | putative NADH:flavin oxidoreductase/NADH oxidase |
| [lma:LMJF_12_0530](https://www.kegg.jp/entry/lma:LMJF_12_0530) | PGI; glucose-6-phosphate isomerase |
| [lma:LMJF_12_0060](https://www.kegg.jp/entry/lma:LMJF_12_0060) | MAR1; ribonuclease mar1 |
| [lma:LMJF_11_1000](https://www.kegg.jp/entry/lma:LMJF_11_1000) | putative pyruvate phosphate dikinase |
| [lma:LMJF_11_0590](https://www.kegg.jp/entry/lma:LMJF_11_0590) | putative 3-methylcrotonoyl-CoA carboxylase beta subunit |
| [lma:LMJF_09_1170](https://www.kegg.jp/entry/lma:LMJF_09_1170) | hypothetical protein |
| [lma:LMJF_09_1120](https://www.kegg.jp/entry/lma:LMJF_09_1120) | mitochondrial RNA binding protein 2 |
| [lma:LMJF_09_0100](https://www.kegg.jp/entry/lma:LMJF_09_0100) | hypothetical protein |
| [lma:LMJF_08_1230](https://www.kegg.jp/entry/lma:LMJF_08_1230) | beta tubulin |
| [lma:LMJF_08_1010](https://www.kegg.jp/entry/lma:LMJF_08_1010) | cathepsin L-like protease |
| [lma:LMJF_08_0960](https://www.kegg.jp/entry/lma:LMJF_08_0960) | hypothetical protein |
| [lma:LMJF_08_0860](https://www.kegg.jp/entry/lma:LMJF_08_0860) | hypothetical protein |
| [lma:LMJF_08_0640](https://www.kegg.jp/entry/lma:LMJF_08_0640) | hypothetical protein |
| [lma:LMJF_07_0800](https://www.kegg.jp/entry/lma:LMJF_07_0800) | flavoprotein subunit-like protein |
| [lma:LMJF_07_0630](https://www.kegg.jp/entry/lma:LMJF_07_0630) | putative vacuolar-type Ca2+-ATPase |
| [lma:LMJF_07_0600](https://www.kegg.jp/entry/lma:LMJF_07_0600) | putative electron transfer flavoprotein-ubiquinone oxidoreductase |
| [lma:LMJF_07_0310](https://www.kegg.jp/entry/lma:LMJF_07_0310) | hypothetical protein |
| [lma:LMJF_06_0880](https://www.kegg.jp/entry/lma:LMJF_06_0880) | putative acyl-coenzyme a dehydrogenase |
| [lma:LMJF_06_0030](https://www.kegg.jp/entry/lma:LMJF_06_0030) | hypothetical protein |
| [lma:LMJF_06_0010](https://www.kegg.jp/entry/lma:LMJF_06_0010) | histone H4 |
| [lma:LMJF_05_0520](https://www.kegg.jp/entry/lma:LMJF_05_0520) | nuclear receptor binding factor-like protein |
| [lma:LMJF_05_0380](https://www.kegg.jp/entry/lma:LMJF_05_0380) | putative microtubule-associated protein |
| [lma:LMJF_05_0350](https://www.kegg.jp/entry/lma:LMJF_05_0350) | TRYR; trypanothione reductase |
| [lma:LMJF_20_1310](https://www.kegg.jp/entry/lma:LMJF_20_1310) | SMP-1; putative calpain-like cysteine peptidase |
| [lma:LMJF_33_0240](https://www.kegg.jp/entry/lma:LMJF_33_0240) | TDR1; thiol-dependent reductase 1 |
| [lma:LMJF_04_0050](https://www.kegg.jp/entry/lma:LMJF_04_0050) | hypothetical protein |
| [lma:LMJF_35_3340](https://www.kegg.jp/entry/lma:LMJF_35_3340) | putative 6-phosphogluconate dehydrogenase,decarboxylating |
| [lma:LMJF_04_0450](https://www.kegg.jp/entry/lma:LMJF_04_0450) | putative calpain-like cysteine peptidase |
| [lma:LMJF_32_2950](https://www.kegg.jp/entry/lma:LMJF_32_2950) | nucleoside diphosphate kinase B |
| [lma:LMJF_04_1230](https://www.kegg.jp/entry/lma:LMJF_04_1230) | ACT; actin |
| [lma:LMJF_23_1086](https://www.kegg.jp/entry/lma:LMJF_23_1086) | SHERP2; small hydrophilic endoplasmic reticulum-associated protein (sherp) |
| [lma:LMJF_04_0240](https://www.kegg.jp/entry/lma:LMJF_04_0240) | hypothetical protein |
