# Supplementary material for: Identification and characterization of yeast SNF1 kinase homologs in Leishmania major
Source: Front Mol Biosci. 2025 Mar 24;12:1567703. doi: 10.3389/fmolb.2025.1567703 (PMC11973601; doi:10.3389/fmolb.2025.1567703)
Supplement: Supplementary file 4 [file Supplementaryfile4.docx]

**Identification and characterization of yeast SNF1 kinase homologs in *Leishmania major***

Gaurav Shoeran^1#^ Namrata Anand ^1^^ Upninder kaur^1^ Kapil Goyal^2^ Rakesh Sehgal^1##*^

1. Department of Medical Parasitology, PGIMER, Chandigarh, India.
2. Department of Virology, PGIMER, Chandigarh, India.

# Present address: College of Pharmacy, University of Kentucky, Lexington, KY, USA.

^Present address:

## present address: Aarupudai Veedu Medical College & Hospital, Puducherry, India

*Corresponding author: Dr. Rakesh Sehgal

Dean, And Professor of Microbiology,

Aarupudai Veedu Medical College & Hospital,

Puducherry, India

Email- [sehgalpgi@gmail.com](mailto:sehgalpgi@gmail.com)

**Supplementary Figure 1**


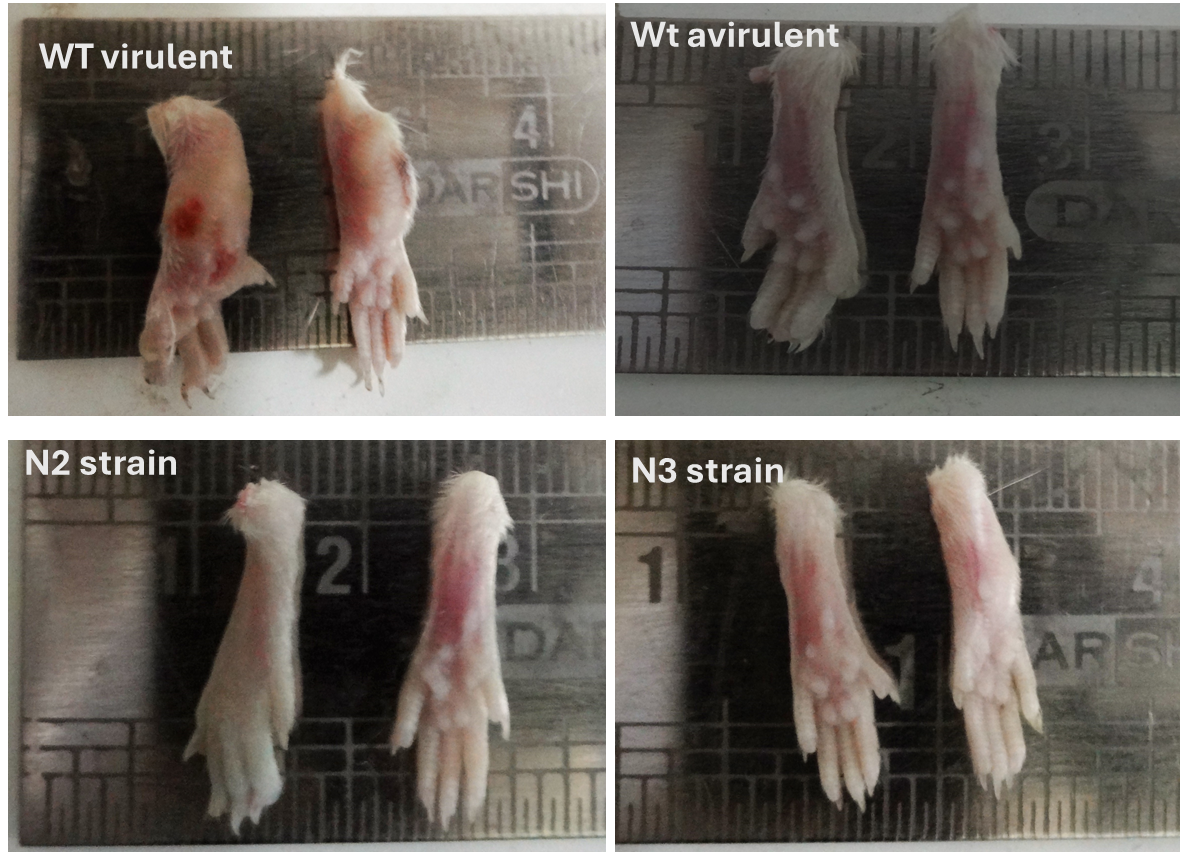


**Supplementary Figure 1.** Food pad images of mice post 1 month of infection.

**Supplementary Figure 2.**


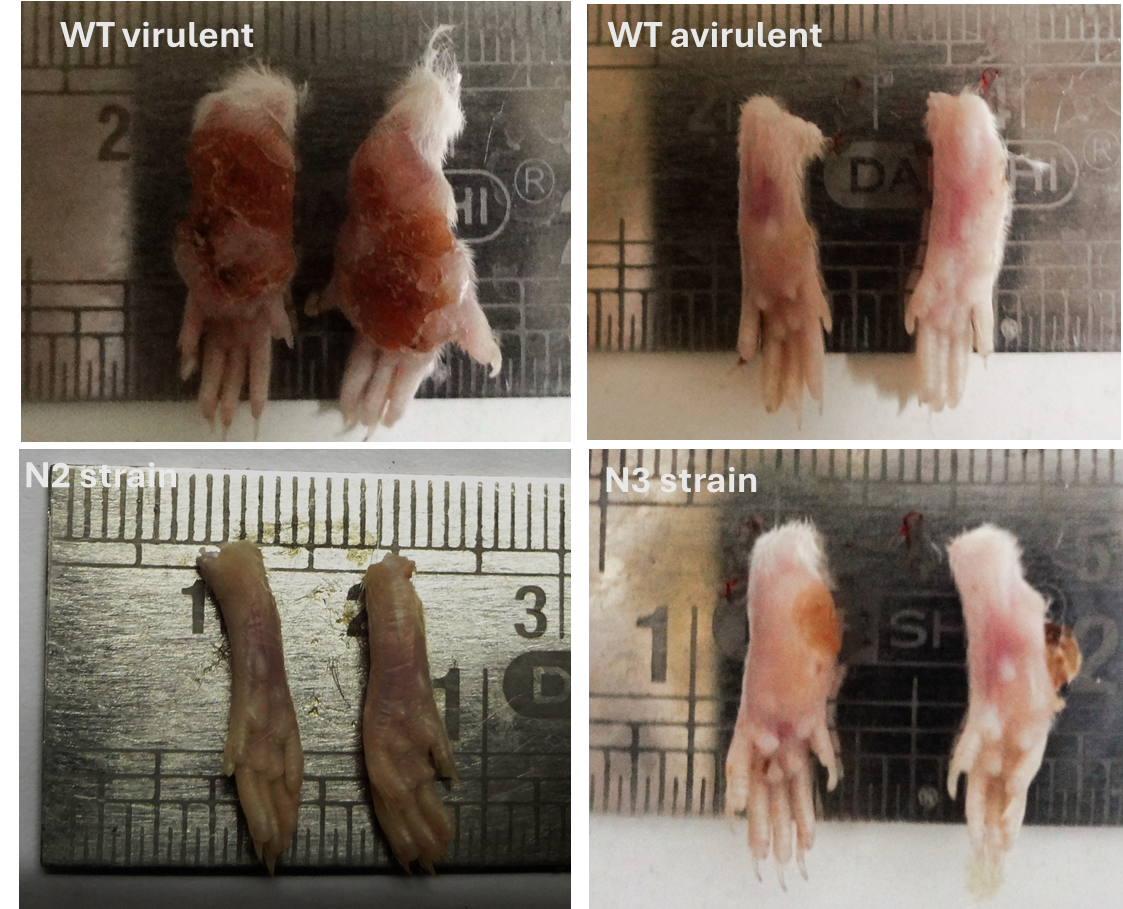


**Supplementary Figure 2.** Representative images of footpad lesions of mice post 2 months of infection.

**Supplementary Figure 3.**

**
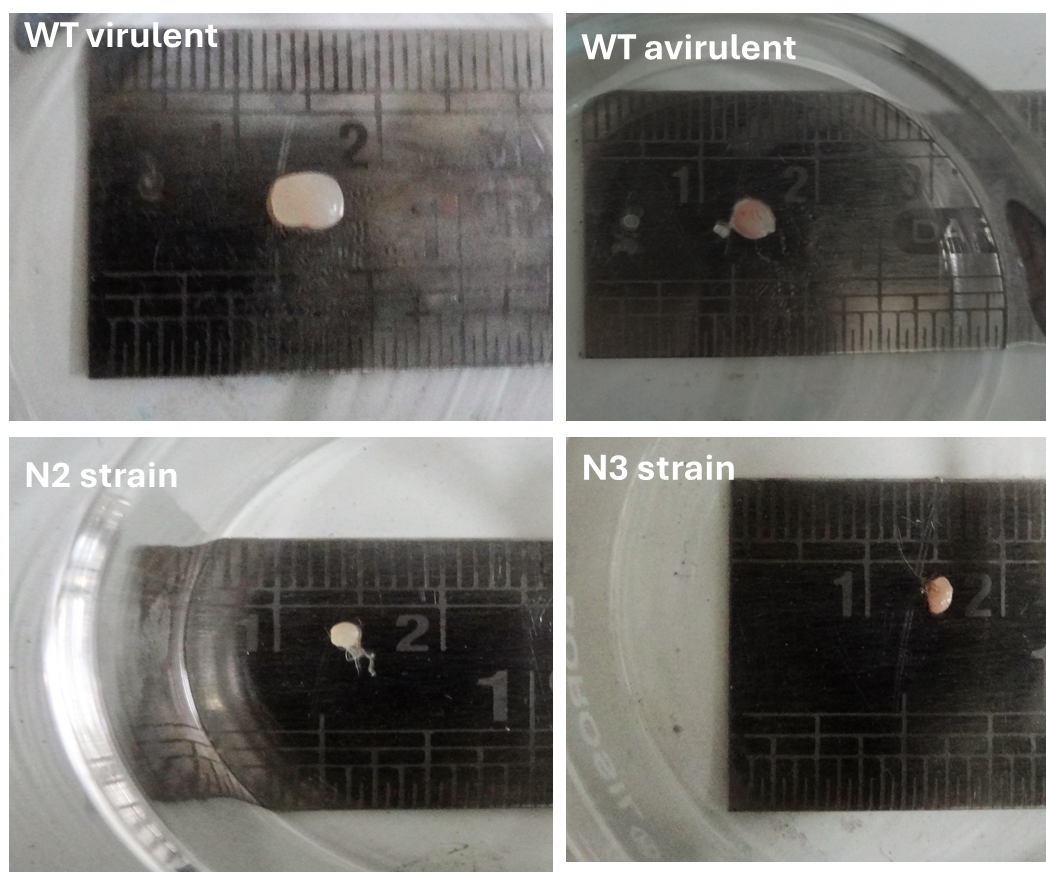
**

**Supplementary Figure 3.** Representative images of lymph node of mice post 1 month infection.

**Supplementary Figure 4.**


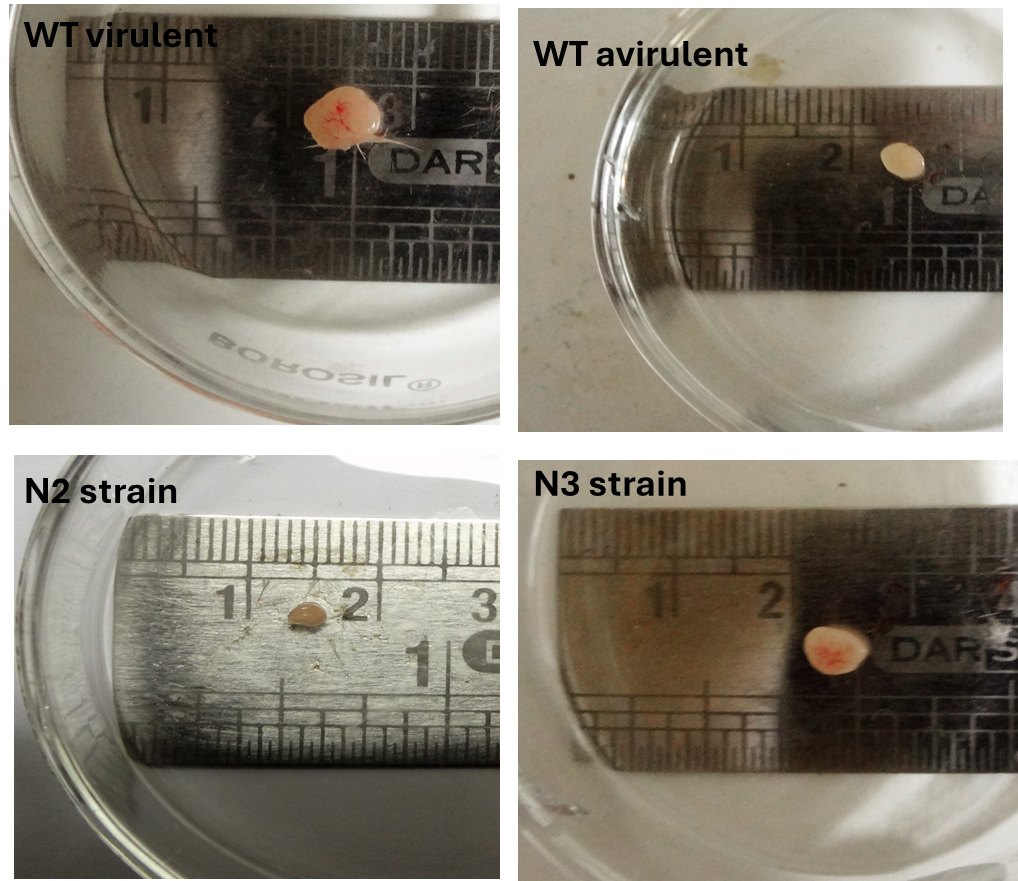


**Supplementary Figure 4.** Representative image of lymph node of mice post 2 months infection.

**Supplementary Figure 5.**

**
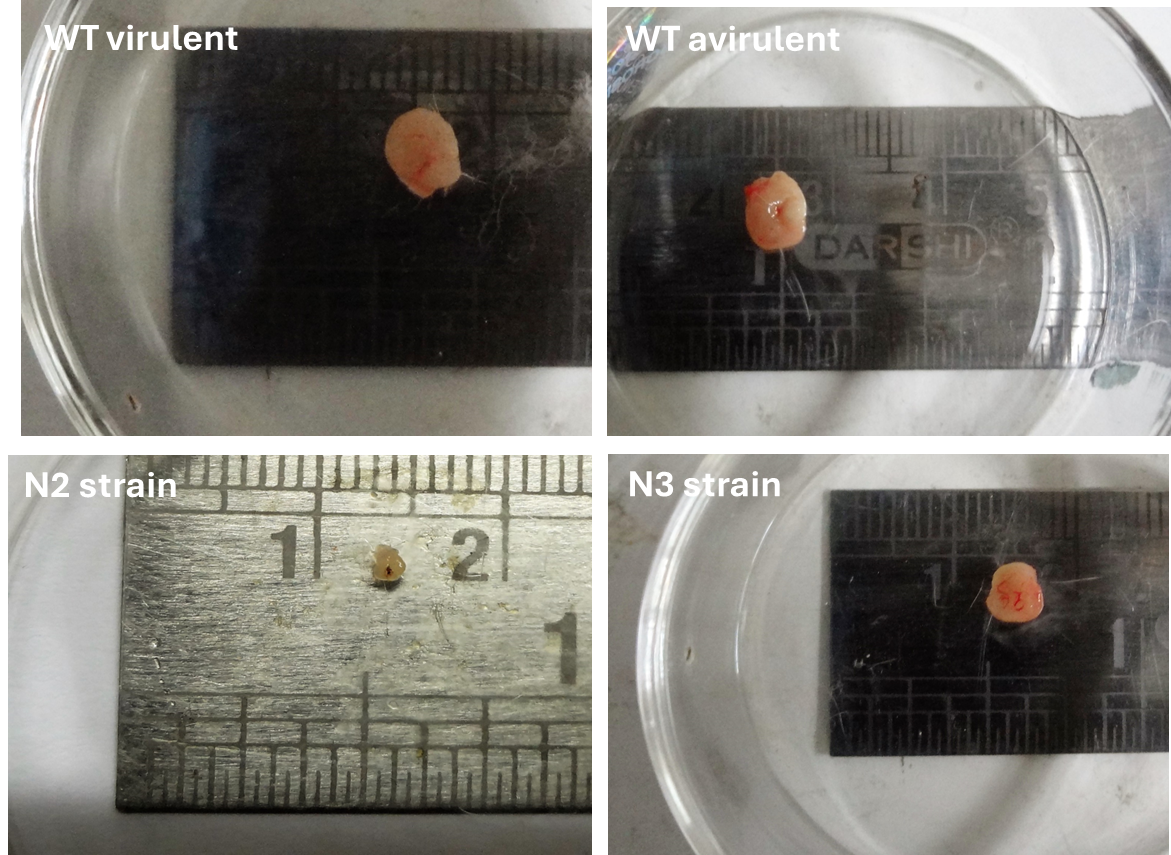
**

**Supplementary Figure 5.** Representative image of lymph node of mice post 3 months infection

**Supplementary Figure 6.**


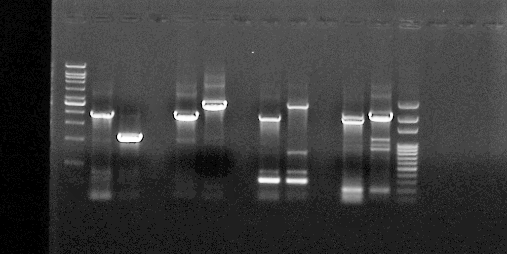


L.D N1 N2 N3 N4 L.D

**Supplementary Figure 6.** Agarose gel electrophoresis of PCR products amplified from different KO strains. N1, N2 and N3 PCR show a single band of about 1800 base pairs which corresponds to the size of inserted cassette. N4 shows two bands, One corresponding to the WT gene fragment and the other to the inserted cassette. This is the original image of the gel and this image has not been used before for any other experimental results or studies before. It just appeared in the reprint of this paper.
